# Glutamate Transporters EAAT2 and EAAT5 Differentially Shape Synaptic Transmission from Rod Bipolar Cell Terminals

**DOI:** 10.1523/ENEURO.0074-22.2022

**Published:** 2022-05-17

**Authors:** Fu-Sheng Tang, He-Lan Yuan, Jun-Bin Liu, Gong Zhang, Si-Yun Chen, Jiang-Bin Ke

**Affiliations:** State Key Laboratory of Ophthalmology, Zhongshan Ophthalmic Center, Sun Yat-sen University, Guangdong Provincial Key Laboratory of Ophthalmology and Visual Science, Guangzhou 510060, China

**Keywords:** amacrine cell, bipolar cell, glutamate transporter, retina, synaptic transmission, temporal resolution

## Abstract

Excitatory amino acid transporters (EAATs) control visual signal transmission in the retina by rapidly removing glutamate released from photoreceptors and bipolar cells (BCs). Although it has been reported that EAAT2 and EAAT5 are expressed at presynaptic terminals of photoreceptors and some BCs in mammals, the distinct functions of these two glutamate transporters in retinal synaptic transmission, especially at a single synapse, remain elusive. In this study, we found that EAAT2 was expressed in all BC types while coexisting with EAAT5 in rod bipolar (RB) cells and several types of cone BCs from mice of either sex. Our immunohistochemical study, together with a recently published literature (Gehlen et al., 2021), showed that EAAT2 and EAAT5 were both located in RB axon terminals near release sites. Optogenetic, electrophysiological and pharmacological analyses, however, demonstrated that EAAT2 and EAAT5 regulated neurotransmission at RB→AII amacrine cell synapses in significantly different ways: EAAT5 dramatically affected both the peak amplitude and kinetics of postsynaptic responses in AIIs, whereas EAAT2 had either relatively small or opposite effects. By contrast, blockade of EAAT1/GLAST, which was exclusively expressed in Müller cells, showed no obvious effect on AII responses, indicating that glutamate uptake by Müller cells did not influence synaptic transmission from RB terminals. Furthermore, we found that temporal resolution at RB→AII synapses was reduced substantially by blockade of EAAT5 but not EAAT2. Taken together, our work reveals the distinct functions of EAAT2 and EAAT5 in signal transmission at RB ribbon synapses.

## Significance Statement

Excitatory amino acid transporters (EAATs) ensure normal information transfer between neurons by rapidly removing glutamate from the synaptic cleft. Although it has been reported that EAAT2 and EAAT5 are expressed at presynaptic terminals of photoreceptors and some bipolar cells (BCs) in the mammalian retina, their functional roles in visual signal transmission remain elusive. Here, we report that EAAT2 and EAAT5 coexist in rod bipolar (RB) cell terminals near release sites, but they influence glutamatergic transmission at RB→AII amacrine cell synapses in significantly different ways, likely via distinct mechanisms. Furthermore, EAAT5 but not EAAT2 plays a predominant role in regulating temporal resolution at RB→AII synapses. Our work therefore reveals the distinct functions of EAAT2 and EAAT5 in signal transmission from RB terminals.

## Introduction

Glutamate, the major excitatory neurotransmitter in the retina, is released primarily by photoreceptors and bipolar cells (BCs), the first-order and second-order retinal neurons, respectively, in the canonical vertical pathway (Masland, 2012; Euler et al., 2014). Both photoreceptors and BCs transmit visual information to postsynaptic neurons through ribbon synapses, named for the existence of a specialized organelle, the synaptic ribbon, which holds synaptic vesicles close to release sites in presynaptic active zones (Matthews and Fuchs, 2010; Cho and von Gersdorff, 2012; Lagnado and Schmitz, 2015). Graded membrane potential changes induce fast and continuous vesicle exocytosis at these ribbon synapses, which will in turn lead to high concentrations of glutamate within the synaptic cleft (Matthews and Fuchs, 2010; Cho and von Gersdorff, 2012; Lagnado and Schmitz, 2015). Excitatory amino acid transporters (EAATs), then, are essential for rapid removal of glutamate from the synaptic cleft to ensure normal synaptic transmission and prevent excitotoxicity (Seal and Amara, 1999; Tzingounis and Wadiche, 2007; Rodríguez-Campuzano and Ortega, 2021).

To date, five subtypes of EAATs, namely, EAAT1–EAAT5, have been identified (Kanai and Hediger, 1992; Pines et al., 1992; Storck et al., 1992; Fairman et al., 1995; Arriza et al., 1997). Previous studies have shown different expression patterns of EAATs in the mammalian retina mainly by immunohistochemistry. In this study, we focused on EAAT2 and EAAT5, both of which served as presynaptic glutamate transporters in retinal neurons as reported previously. EAAT2 (also called GLT-1) is expressed in rod and cone photoreceptors as well as in certain types of BCs in the retinas of various species (Rauen and Kanner, 1994; Rauen et al., 1996, 2004; Eliasof et al., 1998a,b; Harada et al., 1998; Haverkamp and Wässle, 2000; Reye et al., 2002a,b; Fyk-Kolodziej et al., 2004). Interestingly, EAAT5 also has been reported to be expressed in photoreceptors and some BCs, including rod bipolar (RB) cells, in the mouse and rat retinas (Pow and Barnett, 2000; Wersinger et al., 2006). Therefore, EAAT2 and EAAT5 are likely to be co-expressed in photoreceptors. Whether EAAT2 and EAAT5 may coexist in any certain type of BCs is unknown, but an immunohistochemical study in the rat retina has shown that GLT-1B, a splice variant of EAAT2, is expressed in some, but not all, RBs (Reye et al., 2002a), suggesting the coexistence of EAAT2 and EAAT5 in rat retinal RBs.

The distinct roles that EAAT2 and EAAT5 may play in synaptic transmission in the retina, however, remain elusive. While EAAT2 in salamander, goldfish or zebrafish cone photoreceptors has been reported to modify the responses in postsynaptic horizontal cells or BCs (Vandenbranden et al., 1996; Gaal et al., 1998; Niklaus et al., 2017), blockade of EAAT2 does not influence neurotransmission at mouse rod→RB synapses (Hasegawa et al., 2006). Instead, at the same synapses, EAAT5 has been demonstrated to play a predominant role, most likely by rapidly removing glutamate from the synaptic cleft (Hasegawa et al., 2006). EAAT5 located at RB axon terminals, however, is generally thought to function as glutamate-activated anion conductance and provide feedback inhibition to control RB output (Veruki et al., 2006; Wersinger et al., 2006; Ichinose and Lukasiewicz, 2012; Vandenberg and Ryan, 2013; Machtens et al., 2015; Fahlke et al., 2016; Bligard et al., 2020; Lukasiewcz et al., 2021; Kovermann et al., 2022). Additionally, a more recent study with EAAT5 knock-out mice reveals an important role of EAAT5 for improving temporal resolution in the retina, primarily by affecting signal transmission in the rod bipolar pathway (Gehlen et al., 2021). Given that both rods and RBs, the essential neurons in the rod bipolar pathway, express EAAT5, the exact sites that may influence temporal resolution remain to be determined.

In the present work, by conducting single-cell (sc)RNA sequencing (scRNA-seq) and scRT-PCR analyses, we found that EAAT2 was expressed in all mouse retinal BCs and coexisted with EAAT5 in RBs and some cone BC types. Immunohistochemical study further showed that EAAT2 was expressed in RB axon terminals at sites near ribbons. Optogenetic studies of neurotransmission at RB→AII amacrine cell synapses combined with pharmacological analysis revealed that EAAT2 and EAAT5 differentially shaped signal transmission from RB terminals. Furthermore, we demonstrated that blockade of presynaptic EAAT5 but not EAAT2 compromised temporal resolution at RB→AII synapses.

## Materials and Methods

### Animals

All animal procedures were performed in accordance with Sun Yat-sen University animal care committee’s regulations. The transgenic mice, in which channelrhodopsin-2 (ChR2) was expressed predominantly in RBs, were obtained by crossing BAC-Pcp2-IRES-Cre mice (B6.Cg-Tg(Pcp2-cre)^3555Jdhu/J^; Jax 010536) with Ai32 mice (B6.Cg-Gt(ROSA)26Sor^tm32(CAG-COP4*H134R/EYFP)Hze^/J; Jax 012569; Zhang et al., 2005; Ivanova et al., 2010; Madisen et al., 2012). The wild-type C57BL/6J and ChR2 mice of either sex at the ages between 6 and 12 weeks were used in electrophysiological experiments, and wild-type mice of either sex at the ages of postnatal day (P)17 and P42–P84 were used for scRT-PCR and immunohistochemical studies.

### Electrophysiology

Retinal slices were cut at a thickness of 200 μm from light-adapted retinas isolated from both ChR2 and wild-type mice. Retinas were dissected from enucleated eyes in oxygenated (95% O_2_ and 5% CO_2_) Ames’ medium (Sigma), and then embedded in low-melting temperature agarose (Sigma type VIIA, 2–3% in a HEPES buffered saline). Slices were cut on a Leica VT1200s vibratome and stored in oxygenated Ames’ medium at room temperature until use.

Recordings were performed at near-physiological temperature (30–34°C). Slices were perfused continuously (1–2 ml/min) with oxygenated artificial CSF (ACSF) containing (in mm): 119 NaCl, 23 NaHCO_3_, 1.25 NaH_2_PO_4_, 2.5 KCl, 1.15 CaCl_2_, 1.5 MgCl_2_, 10 glucose, 2 Na-Lactate, and 2 Na-Pyruvate. 2-Amino-4-phosphonobutyrate (L-AP4; 5 μm) and (*S*)−1-(2-amino-2-carboxyethyl)−3-(2-carboxy-5-phenylthiophene-3-yl-methyl)−5-methylpyrimidine-2,4-dione (ACET; 1 μm) were included in the ACSF to block all synaptic transmission between photoreceptors and BCs. Picrotoxin (50 μm), 4-imidazoleacetic acid (I4AA; 10 μm) or (1,2,5,6-tetrahydropyridin-4-yl) methylphosphinic acid (TPMPA; 50 μm), and strychnine (0.5 μm) were added to the ACSF to block both GABA and glycine receptors. For AII’s voltage-clamp recordings, ACSF was also supplemented with tetrodotoxin (TTX; 0.5 μm) to block voltage-gated sodium channels.

For voltage-clamp recordings, pipette solution contained (in mm): 95 Cs-methanesulfonate, 20 TEA-Cl, 1 4-AP, 10 HEPES, 8 PO_4_-creatine, 4 ATP-Mg, 0.4 GTP-Na_3_, and 1 BAPTA. For current-clamp recordings, pipette solution contained (in mm): 110 K-gluconate, 5 NaCl, 10 HEPES, 8 PO_4_-creatine, 4 ATP-Mg, 0.4 GTP-Na_3_, and 1 BAPTA. The pH was adjusted to 7.2 with CsOH (for Cs-based pipette solution) or KOH (for K-based pipette solution), and osmolarity to ∼285 mOsm with sucrose. In some cases, Alexa Fluor 594 or Alexa Fluor 488 was included in the pipettes and epifluorescence images were taken to confirm the cell types. Generally, AII was held at −80 mV, and membrane potentials were corrected for junction potentials of ∼−10 mV. Access resistances were <30 ΜΩ for RBs and <20 ΜΩ for AIIs and were compensated by 50–90%. Electrical signals were recorded using MultiClamp 700B amplifiers, sampled at 10–20 kHz and low-pass filtered at 2 kHz by an ITC-18 A/D board (Heka/Instrutech) controlled by software written in Igor Pro 6 (WaveMetrics). Recordings were analyzed in Igor Pro 6.

Dihydrokainic acid (DHK) was obtained from Santa Cruz Biotechnology and Tocris, GT949 was from Absin, UCPH101 was from Abcam, and other chemicals were from Tocris or Sigma-Aldrich. Agents were dissolved in dimethylsulfoxide (DMSO) where appropriate and then diluted into the superfusion solution [with final concentrations of DMSO <0.1% (v/v) in all experiments].

### Optogenetics

ChR2 was activated by a 470-nm high-power LED (Thor Laboratories) directed through a 60× lens to generate a light spot with ∼125-μm diameter. The stimulus paradigm (e.g., light intensities and durations) was controlled by the data acquisition software (Igor Pro 6).

### Fraction of correct responses

To assess the effects of drugs on temporal resolution at RB→AII synapses, light stimulus with a train of 10 consecutive flashes was used to activate ChR2-expressing RBs at various frequencies between 2 and 50 Hz, and the responses in AIIs were recorded and analyzed. The AII response to an individual flash was considered correct when a significant membrane potential change (depolarization in this case) was visible, and the fraction of correct responses (with a value between 0 and 1) was determined as the proportion of correct responses to a stimulus train of 10 flashes.

### Analysis of scRNA-seq datasets

The existing scRNA-seq dataset (GEO accession number: GSE81905) that contains transcriptomes of all mouse retinal BCs (Shekhar et al., 2016) was re-analyzed to determine EAAT subtypes in BCs specifically. To reproduce clustering analysis, the clustering algorithm for the retinal BC data achieved with Vsx2-GFP mice at P17 was implemented and performed using a published R markdown script (Shekhar et al., 2016). The single-cell libraries that contained >10% mitochondrially-derived transcripts were filtered. Batch correction and principal component analysis (PCA) were performed on the cells for which over 500 genes were detected. Among the selected cells, only genes that were present in at least 30 cells and those having over 60 transcripts were considered. Based on the Louvain–Jaccard method (Blondel et al., 2008; Levine et al., 2015), PC scores were used to embed the single cells on a 2D map using t-distributed stochastic neighbor embedding (t-SNE; van der Maaten and Hinton, 2008). The gene expression patterns of EAAT subtypes across BC clusters were shown in dotplots, which illustrated the proportion of different BC clusters (row) that expressed EAAT subtypes (column) using dot size and the average number of EAAT subtype transcripts in specific BC cluster using dot color.

Another existing dataset (GEO accession number: GSE63473), which contained the information about transcriptomes of individual cone and rod photoreceptors from the mouse retinas, was used to examine the transcripts of EAAT subtypes in photoreceptors (Macosko et al., 2015).

### scRT-PCR analysis

The cytoplasmic contents of individual RBs on freshly cut retinal slices were harvested using patch pipettes. Patch pipettes (6–8 MΩ) were pulled from capillaries previously autoclaved and filled with the pipette solution used for current-clamp recordings (above). To better preserve mRNAs, Recombinant Ribonuclease Inhibitor (RRI; 2.5%; catalog #2313A, Clontech) was included in the pipette solution. After establishing whole-cell configuration, the cytoplasm was harvested into the pipette by applying slight negative pressure. The pipette solution without any harvested cell content was used as a negative control.

The harvested cell contents were processed for reverse transcription and sequence-specific amplification using the Single Cell Sequence Specific Amplification kit (catalog #P621-A, Vazyme Biotech). Specific primer pairs (Table 1) were pooled to prepare an Assay Pool (with a final concentration of 0.1 μm for each pair). Then the content of the patch pipette was transferred into PCR tubes with Reaction Mix, Assay Pool, and RT/Taq enzyme. PCR tubes were instantly frozen at −80°C. The tubes were placed on PCR machine following brief centrifugation at 25°C. Thermal cycling conditions were 50°C for 1 h (reverse transcription), 3 min at 95°C (reverse transcriptase inactivation and Taq polymerase activation), followed by preamplification with 20 cycles of 15 s at 95°C (denaturation), 15 min at 60°C (annealing/extension). Subsequently, preamplification products were diluted to 50-fold and re-amplified by another round of PCR with specific primer pairs (with a final concentration of 10 μm for each pair) for targeted genes (Table 1) using Phanta Max Super-Fidelity DNA Polymerase (catalog #P505-d1/d2/d3, Vazyme Biotech). PCR experiments were performed following manufacturer’s instructions with an appropriate annealing temperature (56°C) for 35 cycles. RT-PCR products (10 μl for each one) were electrophoresed through 3% agarose gel, stained with SYBR Safe DNA gel stain (catalog #S33102, Invitrogen) and imaged under UV light excitation. RB identity was confirmed by detection of the *Prkca* gene during scRT-PCR analysis.

**Table 1:** Primers for scRT-PCR analysis

Gene	Protein	Forward primer (5′—3′)	Reverse primer (5′—3′)
*Slc1a2*	EAAT2	CTGATGTGGTCATGTTGATAGCC	AACTGGAGATGATAAGAGGGAGG
*Slc1a7*	EAAT5	TGGCATACTACCTGTGGACTAC	CTTGGTGCGGTACTGTTTGAA
*Prkca*	PKCα	GTTTACCCGGCCAACGACT	GGGCGATGAATTTGTGGTCTT

EAAT2, excitatory amino acid transporter 2; EAAT5, excitatory amino acid transporter 5; PKCα, protein kinase C α.

### Immunohistochemistry

Retinas from both P17 and adult wild-type mice were harvested and fixed for 20 min in 4% PFA. Then retinas were incubated in graded (10%, 20%, and 30%) sucrose in PBS, embedded in OCT, and sectioned vertically with a cryostat (Leica). Thereafter, retinal sections (14-μm thickness) were treated with blocking solution [6% bovine serum albumin (BSA) in 0.1% Triton X-100 in PBS (PBST)] for 2 h at room temperature. After removal of the blocking solution, sections were incubated overnight at 4°C with primary antibodies as follows: rabbit anti-EAAT2 (1:100, catalog #250203, Synaptic Systems; the specificity of this antibody has been verified by EAAT2 knock-out mice as described by the manufacturer), mouse anti-PKCα (1:200, catalog #P5704, Sigma) and guinea pig anti-RIBEYE/CtBP2 (1:1000, catalog #192104, Synaptic Systems). The anti-EAAT2 antibody generated using the peptide immunogen (peptide sequence: LIISSLITGLSGLDAK) can recognize all mouse EAAT2 splice variants. After rinsing, the sections were incubated in corresponding secondary antibodies including Alexa Fluor 568 donkey anti-rabbit (1:200, catalog #A10042, Thermo Fisher Scientific), Alexa Fluor 647 donkey anti-mouse (1:200, catalog #A31571, Thermo Fisher Scientific) and Alexa Fluor 488 goat anti-guinea pig (1:200, catalog #A11073, Thermo Fisher Scientific) for 2 h in darkness at room temperature. All antibodies were diluted in PBST with 3% BSA. Control experiments were conducted either by omission of primary antibodies or by preincubation of primary antibodies with the corresponding immunopeptides. A Zeiss LSM 880 laser-scanning confocal microscope with a Plan-Apochromat 63×/1.4 oil-immersion objective was used for immunofluorescence imaging. Images were edited using Photoshop software (Adobe Systems) and ZEN software (Carl Zeiss).

### Statistical analysis

Prism 8 (GraphPad software) was used to perform the statistical analysis. Original data were used for all the pairwise comparisons; and, for comparisons between different groups, data acquired from each cell were normalized to the value under control condition. Differences between experimental samples were assessed using Student’s *t* test, Welch’s *t* test or Wilcoxon signed-rank test, depending on whether the data passed the normality criterion (Kolmogorov–Smirnov test) or not. Significance was accepted as *p* < 0.05. All data were presented as mean ± SEM.

## Results

### Analysis of scRNA-seq data reveals gene expression of EAAT2 in all mouse retinal BCs and co-expression with EAAT5 in several BC types

**Figure 1.**
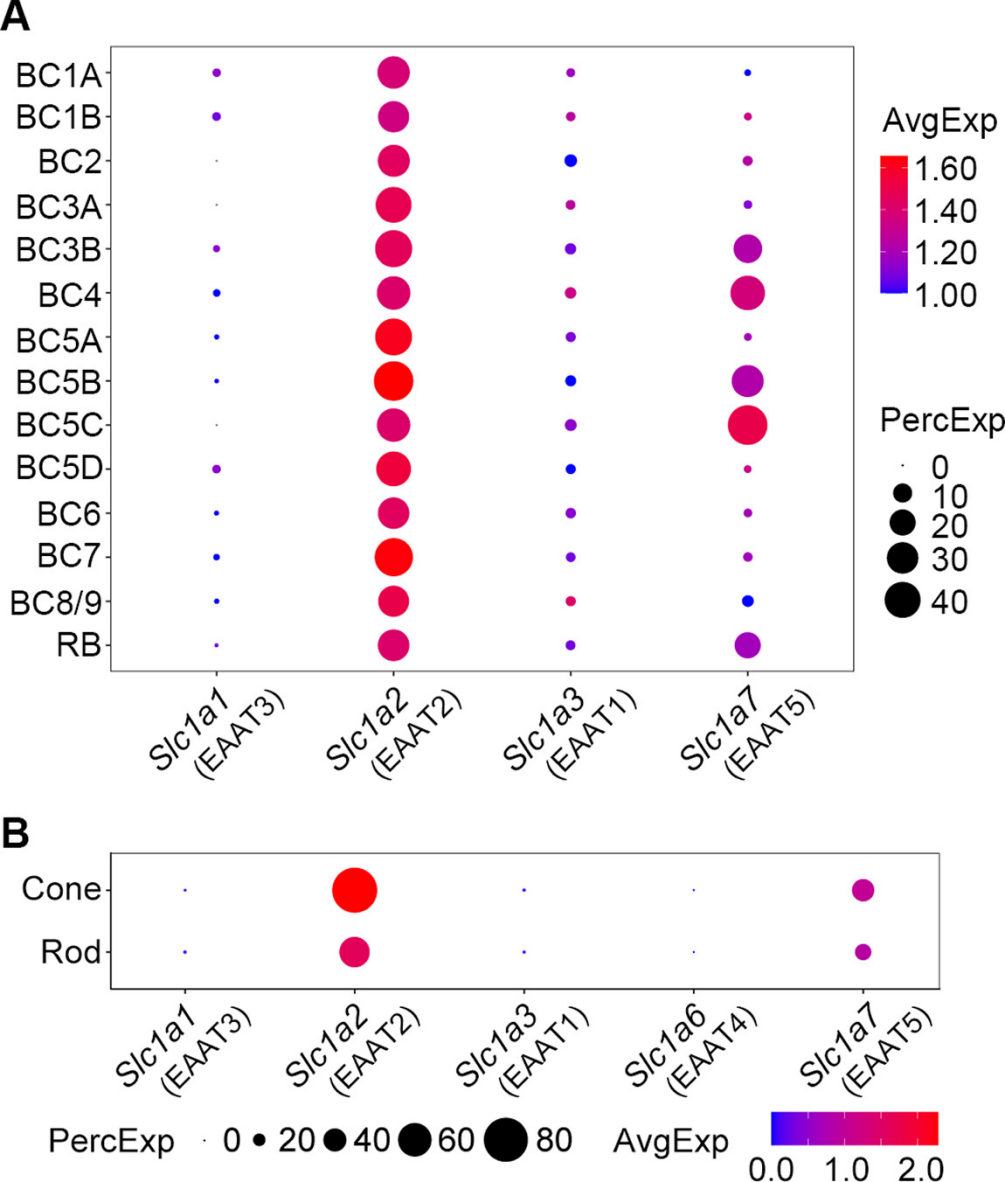
scRNA-seq data analysis reveals gene expression of EAAT2 in all mouse retinal BCs and co-expression with EAAT5 in several BC types. ***A***, Gene expression patterns of EAATs in different types of BCs. The protein that each gene encodes is given in parentheses. The size of each circle represents the percentage of cells in the group (PercExp) in which the gene expression is detected. The color represents the average transcript count in expressing cells (AvgExp). BC, bipolar cell; RB, rod bipolar cell. ***B***, Co-expression of EAAT2 and EAAT5 in cone and rod photoreceptors.

In a recent study, scRNA-seq has been used for molecular classification of mouse retinal BCs, and consequently, the transcriptomes of multiple molecularly defined BC types, including the RB, are available in the Gene Expression Omnibus Database (GEO accession number: GSE81905; Shekhar et al., 2016). We began by re-analyzing this dataset with particular attention to the transcripts of EAAT-related genes. The dotplots in Figure 1 illustrate the average expression level (color) for each BC type and the percentage of EAAT expressing cells (circle size). Surprisingly, we found that EAAT2 transcripts were expressed at relatively high levels in all BC types, while EAAT5 transcripts were expressed at reasonable levels in several types of BCs, more specifically, in RB, BC3B, and BC4 (types 3B and 4 OFF cone BCs, respectively) as well as in BC5B and BC5C (types 5B and 5C ON cone BCs, respectively; Fig. 1*A*). These findings were quite remarkable, because (1) expression of EAAT2 is thought to be restricted to only some BC types and (2) although it is well accepted that EAAT2 and EAAT5 coexist in rod and cone photoreceptors, co-expression of EAAT2 and EAAT5 in any certain type of BCs rarely has been reported in previous studies. As a control for these unexpected findings, we re-analyzed another existing scRNA-seq dataset (GEO accession number: GSE63473), which includes the transcriptomes for mouse retinal rod and cone photoreceptors (Macosko et al., 2015). We found that rod and cone photoreceptors showed much higher levels of mRNAs encoding EAAT2 and EAAT5 than other EAAT-related transcripts (Fig. 1*B*), consistent with previous studies (Haverkamp and Wässle, 2000; Pow and Barnett, 2000; Rauen et al., 2004; Wersinger et al., 2006).

We therefore determined that EAAT2 is expressed in all BC types while coexisting with EAAT5 in RBs as well as in types 3B, 4, 5B, and 5C cone BCs.

### Co-expression of EAAT2 and EAAT5 in mouse retinal RBs is confirmed by scRT-PCR analysis

**Figure 2.**
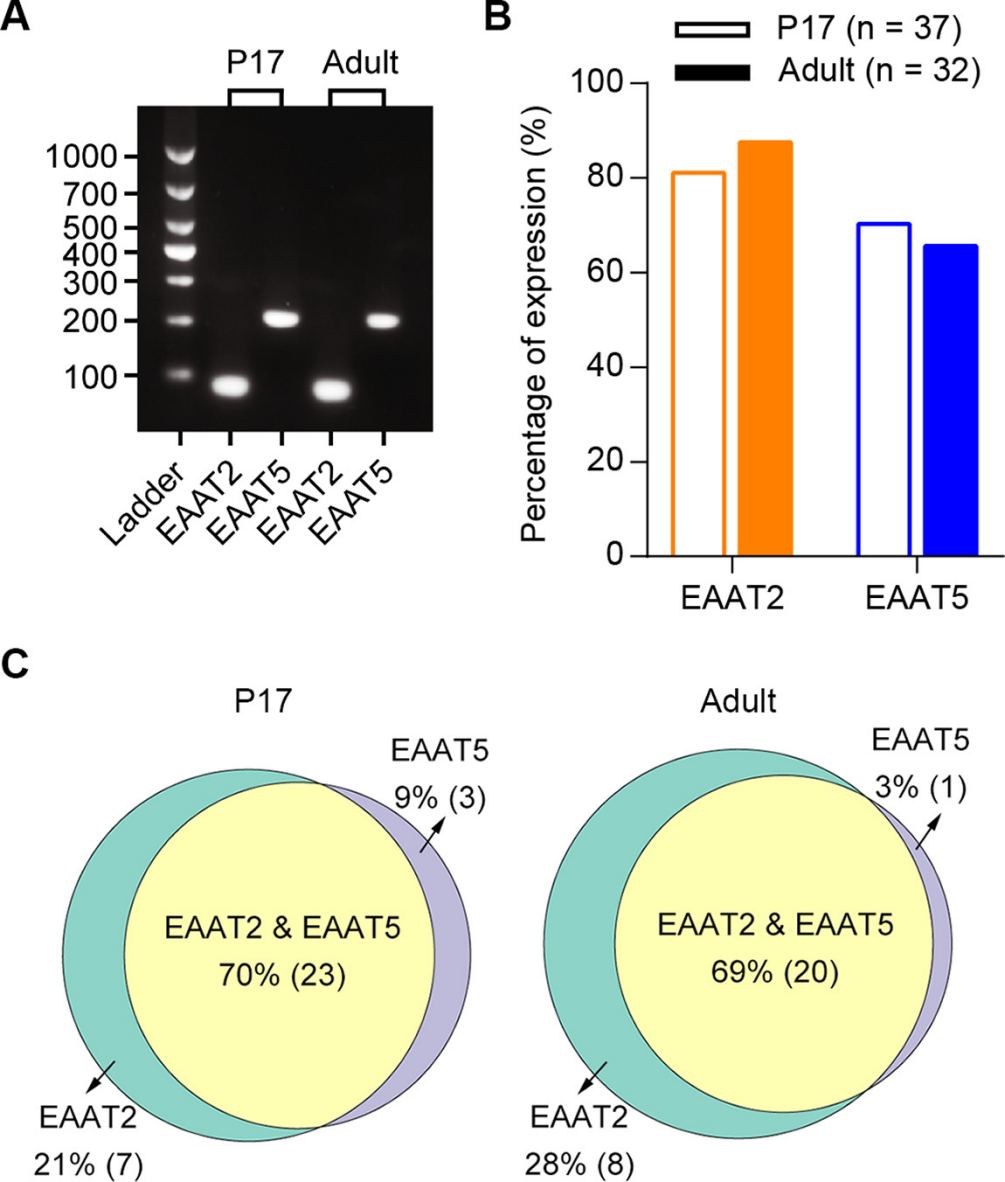
Co-expression of EAAT2 and EAAT5 in mouse retinal RBs is confirmed by scRT-PCR analysis. ***A***, scRT-PCR analyses of EAAT2 and EAAT5 mRNA expression in a single RB cell from a P17 mouse retina and the other RB from an adult mouse retina. Co-expression of EAAT2 and EAAT5 could be seen in both RBs. A ladder with DNA fragments between 100 and 1000 bp is shown on the left. ***B***, The percentages of EAAT2 and EAAT5 expression in individual RBs from both P17 and adult mice. See also Table 2. ***C***, Schematic diagrams illustrating the molecular heterogeneity for EAAT2 and EAAT5 expression in individual RBs from P17 and adult mice. The percentages of either subtype and a combination of EAAT2 and EAAT5 are shown, and the exact cell numbers are given in parentheses. See also Table 3.

Next, we performed a targeted assessment of EAAT2 and EAAT5 transcripts in mouse RBs using scRT-PCR to confirm our observations from scRNA-seq analysis and to examine the potential heterogeneity of EAAT2 and EAAT5 expression in BCs. Since the existing scRNA-seq dataset (GEO accession number: GSE81905) was acquired from mouse retinas at P17 (Shekhar et al., 2016), we conducted scRT-PCR experiments using RBs from both P17 and adult (6- to 12-week-old) mice to determine whether there are developmental changes in EAAT2 and/or EAAT5 expression. Consistent with our scRNA-seq analysis, scRT-PCR detected both EAAT2 and EAAT5 mRNAs in individual RBs from P17 mice (*n* = 37 cells) as well as from adult mice (*n* = 32 cells; Fig. 2*A*,*B*). In general, the mRNA expression of EAAT2 and EAAT5 assessed by scRT-PCR was quite stable from P17 to adulthood (Fig. 2*B*; Table 2).

**Table 2:** Expression of EAAT2 and EAAT5 in P17 and adult mouse retinal RBs

	EAAT2	EAAT5
P17 mice		
Total RB number	37	37
Positive RB number	30	26
Negative RB number	7	11
Percentage of expression	81.08%	70.27%
Adult mice		
Total RB number	32	32
Positive RB number	28	21
Negative RB number	4	11
Percentage of expression	87.50%	65.63%

EAAT2, excitatory amino acid transporter 2; EAAT5, excitatory amino acid transporter 5; P17, postnatal day 17; RB, rod bipolar cell.

**Table 3:** Molecular heterogeneity of EAAT2 and EAAT5 in individual mouse retinal RBs

	P17 mice		Adult mice	
	RB number	Percentage	RB number	Percentage
EAAT2 alone	7	21.21%	8	27.59%
EAAT5 alone	3	9.09%	1	3.45%
EAAT2 and EAAT5	23	69.70%	20	68.97%
Total	33		29	

EAAT2, excitatory amino acid transporter 2; EAAT5, excitatory amino acid transporter 5; P17, postnatal day 17; RB, rod bipolar cell.

It is noteworthy that although the expression profiles of EAAT2 and EAAT5 varied among individual cells within the population of RBs (Fig. 2*C*; Table 3), most RBs expressed both EAAT2 and EAAT5: the specific combination of EAAT2 and EAAT5 was found in ∼70% and 69% of EAAT2/EAAT5-expressing RBs from P17 and adult mice, respectively; this is in line with the scRNA-seq analysis (Fig. 1*A*).

In conclusion, it would seem that the majority of RBs co-express EAAT2 and EAAT5.

### EAAT2 is located near ribbons in the axon terminals of RBs

Previous studies have shown in rat and mouse retinas that EAAT5 is located in RB axon terminals (Pow and Barnett, 2000; Wersinger et al., 2006). In addition, a more recent study has reported that EAAT5 is expressed in a punctate manner near the release sites in the axon terminals of mouse RBs (Gehlen et al., 2021). To examine the subcellular localization and potential developmental changes of EAAT2 proteins, we performed fluorescent immunohistochemical labeling of EAAT2, PKCα (a specific cell marker of RBs), and RIBEYE (a ribbon-specific protein) in both P17 and adult mouse retinal sections. Consistent with the scRT-PCR results, no significant differences in the expression patterns of EAAT2 were observed between P17 and adult retinas. Hence, for simplicity, we present only the results from the adult retinas here.

**Figure 3.**
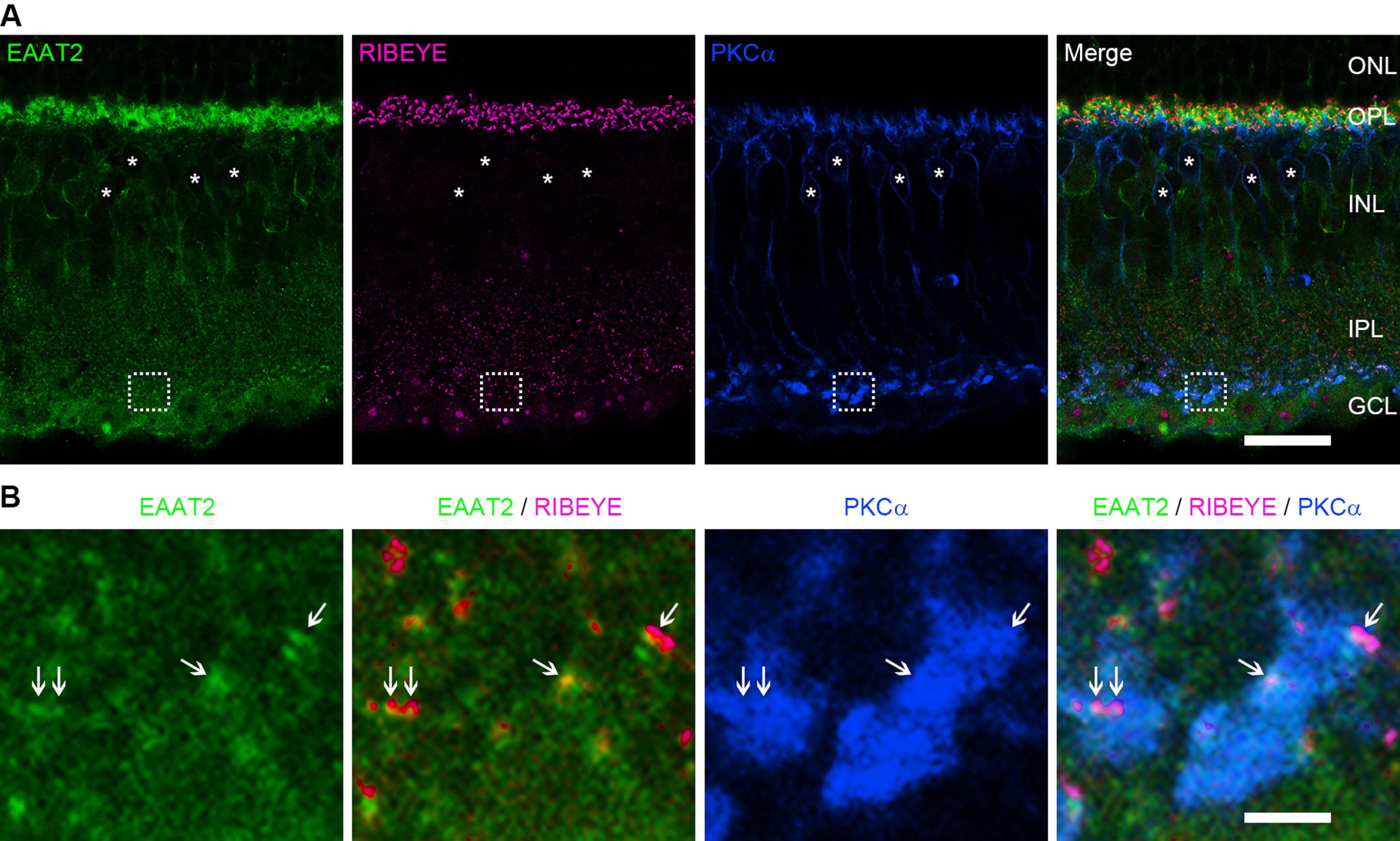
EAAT2 is located near ribbons in the axon terminals of RBs. ***A***, Confocal images showing immunofluorescence triple labeling of EAAT2 (green), RIBEYE (magenta), and PKCα (blue) in a frozen mouse retinal section. EAAT2 was expressed strongly in the OPL, and moderately in the INL, IPL, and GCL. Note that, in the INL, EAAT2 was expressed in the somata of some cone bipolar cells but not RB cells labeled by PKCα (asterisks). ONL, outer nuclear layer; OPL, outer plexiform layer; INL, inner nuclear layer; IPL, inner plexiform layer; GCL, ganglion cell layer. Scale bar: 20 μm. ***B***, Magnification of the images in the dashed line frames of ***A***. EAAT2 was expressed in RB axon terminals at sites near ribbons (arrows). Scale bar: 2.5 μm.

EAAT2 was found to be expressed most prominently in the outer plexiform layer (OPL), moderately in the inner nuclear layer (INL) and throughout the inner plexiform layer (IPL) in a punctate manner (Fig. 3*A*). In the INL, immunolabeling for EAAT2 was observed on the cell membrane of some BCs which were not labeled by PKCα, indicating that EAAT2 is expressed in the somata of some cone BCs, but not RBs (Fig. 3*A*). Remarkably, we found that EAAT2 was located in the axon terminals of RBs at sites near ribbons (Fig. 3*B*), suggesting that EAAT2 might play an important role in regulating synaptic transmission from RB terminals.

### EAAT2 regulates signal transmission at RB→AII ribbon synapses

**Figure 4.**
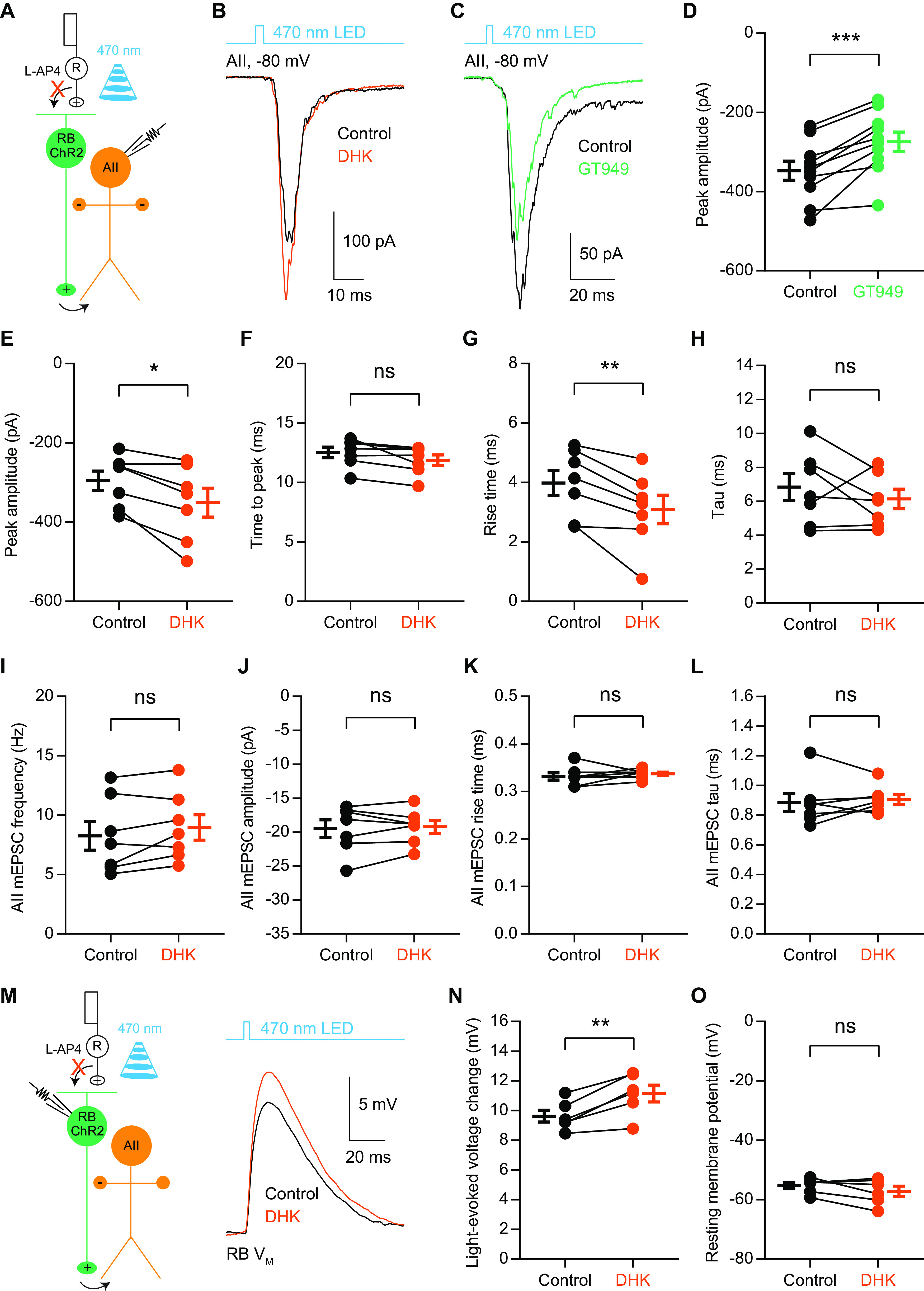
EAAT2 regulates signal transmission at RB→AII ribbon synapses. ***A***, A schematic diagram showing the optogenetic study of neurotransmission between RBs and AII amacrine cells. ChR2 was expressed predominantly in RBs by cre-dependent recombination in adult Pcp2-cre::Ai32 mouse retinas. With all the synaptic transmission between photoreceptors and BCs is blocked pharmacologically, brief flashes of 470-nm LED light can directly activate ChR2+ RBs and induce postsynaptic responses in AIIs, which mainly reflect neurotransmitter release from RBs. The electrical coupling between ChR2+ ON cone bipolar cells and AIIs is negligible under this experimental condition (Liang et al., 2021). R, rod. ***B***, The EPSCs recorded in AIIs, which were evoked by 470-nm LED light stimulation, were enhanced by 200 μm DHK, a selective EAAT2 blocker. V_hold_ = −80 mV. ***C***, The ChR2-evoked EPSCs were reduced by 10 μm GT949, a positive allosteric modulator of EAAT2. ***D***, ***E***, Summary data showing the effects of GT949 (*n* = 10) and DHK (*n* = 7) on the peak amplitude of AII EPSCs. ***F***, DHK reduced the time to peak of EPSCs slightly, but not significantly (*n* = 7, *p* = 0.0531). ***G***, DHK reduced the rise time of EPSCs (*n* = 7). ***H***, DHK did not change the decay time (tau) of EPSCs (*n* = 7). ***I–L***, DHK did not affect the frequency, amplitude, rise time, or tau of mEPSCs recorded in AIIs (*n* = 7). mEPSCs, miniature EPSCs. ***M***, The voltage changes in ChR2+ RBs, which were evoked by brief flashes of 470-nm LED light, were increased by 200 μm DHK. ***N***, DHK increased the voltage changes in RBs evoked by light flashes (*n* = 6). ***O***, DHK did not influence the resting membrane potentials of RBs (*n* = 6). The data were represented as mean ± SEM. Wilcoxon signed-rank test or Student’s *t* test was used where appropriate. *p < 0.05, **p < 0.01; ns, not significantly different. See also Table 4.

Our immunohistochemical study, together with a recently published literature (Gehlen et al., 2021), indicate that EAAT2 and EAAT5 are both located near the release sites in the axon terminals of mouse RBs. The distinct roles that EAAT2 and EAAT5 may play in signal transmission at RB→AII synapses, however, remains elusive. It has been shown recently that, by expressing the light-sensitive cation channel ChR2 predominantly in RBs using cre-mediated recombination in the Pcp2-cre::Ai32 mouse retina, optogenetic stimulation of neurotransmission at RB→AII synapses could be stable over long periods (>20 min; Liang et al., 2021). To determine whether EAAT2 and EAAT5 contribute to synaptic transmission between RBs and AIIs, we blocked all the transmission from photoreceptors to second-order retinal neurons (i.e., BCs and horizontal cells) with the mGluR6 agonist L-AP4 (5 μm) and the kainate receptor antagonist ACET (1 μm) and used brief flashes (2–10 ms) of 470-nm LED light to activate RBs; optogenetically-evoked EPSCs and miniature EPSCs (mEPSCs) recorded in AIIs under this condition reflect evoked and spontaneous release from RBs, respectively (Fig. 4*A*; Liang et al., 2021). As a control, no visible light-evoked EPSCs could be detected in AIIs from wild-type mice under the same experimental condition (*n* = 2; data not shown).

**Table 4:** The effects of EAAT2-related drugs on AII responses and RB membrane potentials

	Data	Data structure	Type of test	Power	Mean ± SEM	Number of cells
	Effect of DHK on AII EPSC amplitude (unit: pA)					
	Control	Normal distribution			−295.40 ± 24.45	7
	200 μm DHK	Normal distribution			−350.70 ± 36.26	7
a	Control vs DHK		Paired Student’s *t* test	*p* = 0.0111		7
	Effect of GT949 on AII EPSC amplitude (unit: pA)					
	Control	Normal distribution			−347.50 ± 24.07	10
	10 μm GT949	Normal distribution			−274.40 ± 24.73	10
b	Control vs GT949		Paired Student’s *t* test	*p* = 0.0004		10
	Relative effects of DHK and GT949 on AII EPSC amplitude					
	Control	Non-normal distribution			1.00 ± 0.00	
	200 μm DHK	Normal distribution			1.18 ± 0.04	7
	10 μm GT949	Normal distribution			0.78 ± 0.03	10
c	Control vs DHK		Wilcoxon signed-rank test	*p* = 0.0313		7
d	Control vs GT949		Wilcoxon signed-rank test	*p* = 0.0020		10
	Effect of DHK on AII EPSC time to peak (unit: ms)					
	Control	Normal distribution			12.53 ± 0.44	7
	200 μm DHK	Normal distribution			11.87 ± 0.44	7
e	Control vs DHK		Paired Student’s *t* test	*p* = 0.0531		7
	Effect of DHK on AII EPSC rise time (unit: ms)					
	Control	Normal distribution			3.98 ± 0.43	7
	200 μm DHK	Normal distribution			3.09 ± 0.48	7
f	Control vs DHK		Paired Student’s *t* test	*p* = 0.0050		7
	Effect of DHK on AII EPSC tau (unit: ms)					
	Control	Normal distribution			6.73 ± 0.80	7
	200 μm DHK	Normal distribution			6.03 ± 0.58	7
g	Control vs DHK		Paired Student’s *t* test	*p* = 0.3519		7
	Effect of DHK on AII mEPSC frequency (unit: Hz)					
	Control	Normal distribution			8.25 ± 1.20	7
	200 μm DHK	Normal distribution			8.97 ± 1.07	7
h	Control vs DHK		Paired Student’s *t* test	*p* = 0.1120		7
	Effect of DHK on AII mEPSC amplitude (unit: pA)					
	Control	Normal distribution			−19.47 ± 1.30	7
	200 μm DHK	Normal distribution			−19.23 ± 0.95	7
i	Control vs DHK		Paired Student’s *t* test	*p* = 0.6892		7
	Effect of DHK on AII mEPSC rise time (unit: ms)					
	Control	Normal distribution			0.33 ± 0.01	7
	200 μm DHK	Non-normal distribution			0.34 ± 0.00	7
j	Control vs DHK		Wilcoxon signed-rank test	*p* = 0.3750		7
	Effect of DHK on AII mEPSC tau (unit: ms)					
	Control	Non-normal distribution			0.88 ± 0.06	7
	200 μm DHK	Normal distribution			0.90 ± 0.03	7
k	Control vs DHK		Wilcoxon signed-rank test	*p* = 0.5781		7
	
	Effect of DHK on RB light-evoked voltage change (unit: mV)					
	Control	Normal distribution			9.62 ± 0.39	6
	200 μm DHK	Normal distribution			11.14 ± 0.56	6
l	Control vs DHK		Paired Student’s *t* test	*p* = 0.0036		6
	Effect of DHK on RB resting membrane potential (unit: mV)					
	Control	Normal distribution			−55.28 ± 1.04	6
	200 μm DHK	Normal distribution			−57.21 ± 1.76	6
m	Control vs DHK		Paired Student’s *t* test	*p* = 0.1574		6

DHK, a selective EAAT2 blocker; GT949, a positive allosteric modulator of EAAT2; AII, AII amacrine cell; RB, rod bipolar cell.

Evoked EPSCs recorded in AIIs were bidirectionally regulated by application of 200 μm DHK, a selective EAAT2 blocker, and by application of 10 μm GT949, a positive allosteric modulator of EAAT2 (Kortagere et al., 2018): DHK increased the peak amplitude of EPSCs (118 ± 4% of control; *n* = 7), while GT949 reduced it (78 ± 3% of control; *n* = 10; Fig. 4*B–E*; Table 4). We also examined the effect of 500 μm DHK on AII EPSCs, and found no significant differences in the relative effect on the peak amplitude between 200 and 500 μm DHK (500 μm DHK, 122 ± 9% of control, *n* = 5; 200 vs 500 μm DHK, Welch’s *t* test, *p* = 0.7771), indicating that EAAT2 is blocked completely by 200 μm DHK under this experimental condition. Therefore, 200 μm DHK was used in all subsequent experiments related to EAAT2. DHK also changed the kinetics of EPSCs (Table 4): the time to peak seemed to be reduced slightly, but not significantly (Fig. 4*F*), and accordingly, the rise time was decreased (Fig. 4*G*); however, the tau was not changed (Fig. 4*H*).

DHK, however, had no significant effects on the frequency, amplitude, rise time, or tau of mEPSCs recorded in AIIs (Fig. 4*I–L*; Table 4), indicating that blockade of EAAT2 does not influence spontaneous release from RB terminals and DHK has no effects on the biophysical properties of postsynaptic AMPA receptors on AIIs.

It has been reported in the rat retina that in the presence of TBOA, a nonselective EAAT blocker, RBs are more depolarized in response to a positive current injection and the rise time of the EPSCs recorded in AIIs is reduced when compared with the control condition; the effects of TBOA are thought to result from blocking EAAT5-mediated anion conductance in RB axon terminals (Veruki et al., 2006). To determine whether blockade of EAAT2 may influence the excitability of RBs, we tested the effect of DHK on membrane potential changes recorded in ChR2+ RBs which were evoked by brief flashes of light. Application of 200 μm DHK increased the light-evoked voltage changes in RBs (Fig. 4*M*,*N*; Table 4), but did not affect the resting membrane potentials (Fig. 4*O*; Table 4).

Taken together, these results demonstrated that blockade of EAAT2 in RB axon terminals enhances and accelerates synaptic transmission at RB→AII synapses, likely by increasing the light-evoked membrane potential changes in RBs.

### Pharmacological blockade of all EAATs has a significant effect on signal transmission at RB→AII synapses

Since EAAT5 has also been reported to be present in the axon terminals of mouse RBs (Wersinger et al., 2006), one might want to know whether it may play a different role in regulating signal transmission at RB→AII synapses. The fact that no selective blockers for EAAT5 are available, however, makes targeted pharmacological manipulation of EAAT5 impractical. Therefore, we compared the effects of DHK (blocking EAAT2 only) and TBOA (blocking all EAATs) on ChR2-evoked EPSCs in AIIs to determine whether other EAATs (e.g., EAAT5 in RBs and EAAT1 in Müller cells) may be involved in regulating neurotransmission at RB→AII synapses.

**Figure 5.**
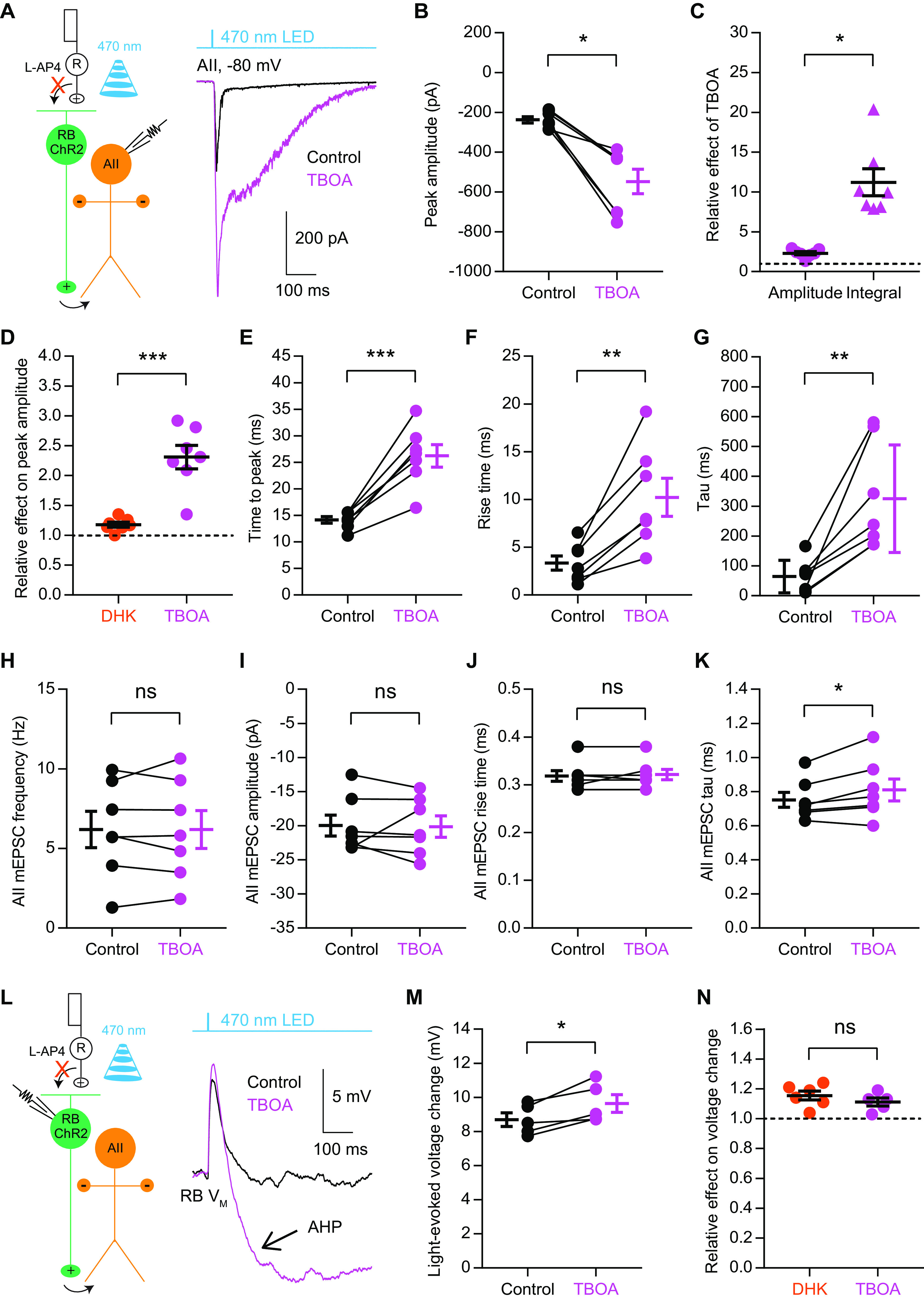
Pharmacological blockade of all EAATs has a significant effect on signal transmission at RB→AII synapses. ***A***, The EPSCs recorded in AII amacrine cells, which were evoked by activating ChR2+ RBs with 470-nm LED light stimulation, were enhanced by 50 μm TBOA, a nonselective blocker of all EAATs. V_hold_ = −80 mV. R, rod; RB, rod bipolar cell; ChR2, channelrhodopsin-2. ***B***, TBOA increased the peak amplitude of ChR2-evoked EPSCs (*n* = 7). ***C***, The relative effects of TBOA on the peak amplitude and current integral of AII EPSCs (*n* = 7). The peak amplitudes/integrals were normalized to the peak amplitude/integral under control condition in each cell before averaging across cells. ***D***, Comparison of the relative effects of DHK (*n* = 7), a selective EAAT2 blocker, and TBOA (*n* = 7) on the peak amplitude of AII EPSCs. ***E–G***, TBOA changed the time to peak, rise time and tau of EPSCs (*n* = 7). ***H–K***, TBOA did not influence the frequency, amplitude, or rise time of mEPSCs recorded in AIIs while increasing the tau slightly (*n* = 7). mEPSCs, miniature EPSCs. ***L***, The voltage changes in ChR2+ RBs, which were evoked by brief flashes of 470-nm LED light, were increased by 50 μm TBOA. Note that, in the presence of TBOA, a large, long-lasting AHP (arrow) could be recorded in each RB following the light-evoked depolarization. ***M***, TBOA increased the initial voltage changes in RBs evoked by light flashes (*n* = 5). ***N***, Comparison of the relative effects of DHK (*n* = 6) and TBOA (*n* = 5) on light-evoked voltage changes in RBs. The data were represented as mean ± SEM. Wilcoxon signed-rank test or Student’s *t* test was used where appropriate. *p < 0.05, **p < 0.01, ***p < 0.001; ns, not significantly different. See also Table 5.

Remarkably, ChR2-evoked EPSCs in AIIs were enhanced substantially by application of 50 μm TBOA (Fig. 5*A*). Unlike DHK, which increased the transient component of EPSCs only (Fig. 4*B*), TBOA enhanced both the transient and sustained components (Fig. 5*A*,*B*; Table 5), with a much stronger effect on the latter (Fig. 5*C*; Table 5). When compared with DHK, TBOA had a greater effect on the transient component (Fig. 5*D*; Table 5). Furthermore, TBOA increased the time to peak, rise time and tau of EPSCs (Fig. 5*E–G*; Table 5), suggesting that, in the presence of TBOA, a huge amount of glutamate accumulating in the synaptic cleft might diffuse to the postsynaptic sites and activate glutamate receptors asynchronously; and accordingly, glutamate might diffuse out of the synaptic cleft very slowly under this experimental condition. Apparently, pharmacological blockade of all EAATs had a much stronger effect on AII EPSCs than blocking EAAT2 only, indicating that EAATs other than EAAT2 also are involved in regulating synaptic transmission at RB→AII synapses.

**Table 5:** The effects of TBOA on AII responses and RB membrane potentials

	Data	Data structure	Type of test	Power	Mean ± SEM	Number of cells
	Effect of TBOA on AII EPSC amplitude (unit: pA)					
	Control	Normal distribution			−237.40 ± 16.21	7
	50 μm TBOA	Non-normal distribution			−547.20 ± 61.50	7
a	Control vs TBOA		Wilcoxon signed-rank test	*p* = 0.0156		7
	Relative effects of TBOA on AII EPSC amplitude and integral					
	Control	Non-normal distribution			1.00 ± 0.00	7
	50 μm TBOA (amplitude)	Normal distribution			2.31 ± 0.20	7
	50 μm TBOA (integral)	Non-normal distribution			11.22 ± 1.70	7
b	Control vs TBOA (amplitude)		Wilcoxon signed-rank test	*p* = 0.0156		7
c	Control vs TBOA (integral)		Wilcoxon signed-rank test	*p* = 0.0156		7
d	TBOA (amplitude) vs TBOA (integral)		Wilcoxon signed-rank test	*p* = 0.0156		7
	Relative effect of DHK on AII EPSC amplitude					
	200 μm DHK	Normal distribution			1.18 ± 0.04	7
e	DHK vs TBOA (amplitude)		Unpaired Student’s *t* test	*p* = 0.0001		
	Effect of TBOA on AII EPSC time to peak (unit: ms)					
	Control	Normal distribution			14.16 ± 0.62	7
	50 μm TBOA	Normal distribution			26.21 ± 2.12	7
f	Control vs TBOA		Paired Student’s *t* test	*p* = 0.0004		7
	Effect of TBOA on AII EPSC rise time (unit: ms)					
	Control	Normal distribution			3.34 ± 0.75	7
	50 μm TBOA	Normal distribution			10.23 ± 1.99	7
g	Control vs TBOA		Paired Student’s *t* test	*p* = 0.0032		7
	Effect of TBOA on AII EPSC tau (unit: ms)					
	Control	Non-normal distribution			64.54 ± 20.61	7
	50 μm TBOA	Normal distribution			325 ± 68.02	7
h	Control vs TBOA		Paired Student’s *t* test	*p* = 0.0050		7
	Effect of TBOA on AII mEPSC frequency (unit: Hz)					
	Control	Normal distribution			6.19 ± 1.14	7
	50 μm TBOA	Normal distribution			6.19 ± 1.19	7
i	Control vs TBOA		Paired Student’s *t* test	*p* = 0.9854		7
	Effect of TBOA on AII mEPSC amplitude (unit: pA)					
	Control	Normal distribution			−19.98 ± 1.55	7
	50 μm TBOA	Normal distribution			−20.13 ± 1.57	7
j	Control vs TBOA		Paired Student’s *t* test	*p* = 0.8856		7
	Effect of TBOA on AII mEPSC rise time (unit: ms)					
	Control	Non-normal distribution			0.32 ± 0.01	7
	50 μm TBOA	Normal distribution			0.32 ± 0.01	7
k	Control vs TBOA		Wilcoxon signed-rank test	*p* > 0.9999		7
	Effect of TBOA on AII mEPSC tau (unit: ms)					
	Control	Normal distribution			0.75 ± 0.04	7
	50 μm TBOA	Normal distribution			0.81 ± 0.06	7
l	Control vs TBOA		Paired Student’s *t* test	*p* = 0.0392		7
	Effect of TBOA on RB light-evoked voltage change (unit: mV)					
	Control	Normal distribution			8.69 ± 0.39	5
	50 μm TBOA	Normal distribution			9.64 ± 0.51	5
m	Control vs TBOA		Paired Student’s *t* test	*p* = 0.0192		5
	Effect of TBOA on RB resting membrane potential (unit: mV)					
	Control	Normal distribution			−52.92 ± 2.01	5
	50 μm TBOA	Normal distribution			−52.91 ± 2.95	5
n	Control vs TBOA		Paired Student’s *t* test	*p* = 0.9931		5
	Relative effects of DHK and TBOA on RB light-evoked voltage change					
	200 μm DHK	Normal distribution			1.16 ± 0.03	6
	50 μm TBOA	Normal distribution			1.11 ± 0.03	5
o	DHK vs TBOA		Unpaired Student’s *t* test	*p* = 0.3226		

TBOA, a non-selective EAAT blocker; DHK, a selective EAAT2 blocker; AII, AII amacrine cell; RB, rod bipolar cell.

By contrast, TBOA did not influence the frequency, amplitude, or rise time of mEPSCs recorded in AIIs (Fig. 5*H–J*; Table 5), indicating that blockade of all EAATs does not affect spontaneous release from RB terminals. Interestingly, TBOA seemed to increase the tau of mEPSCs slightly (Fig. 5*K*; Table 5). The relative effect of TBOA on the tau of mEPSCs (107 ± 3% of control, *n* = 7), however, was much smaller than its effect on the tau of EPSCs (911 ± 317% of control; *n* = 7), and thus could not account for the large effect of TBOA on synaptic transmission at RB→AII synapses.

To determine to what extent blockade of both EAAT2 and EAAT5 may influence the excitability of RBs, we tested the effect of TBOA on membrane potential changes in ChR2+ RBs evoked by brief flashes of light. Similar to our observations with DHK (Fig. 4*L*,*M*), application of 50 μm TBOA increased the light-evoked voltage changes in RBs (Fig. 5*L*,*M*; Table 5), but did not affect the resting membrane potentials (Table 5). It is noteworthy that in the presence of TBOA, a large afterhyperpolarization (AHP) could be observed following the fast light-evoked depolarization of each recorded RB (Fig. 5*L*). The relative effects of DHK and TBOA on the fast depolarizations of RBs were not significantly different (Fig. 5*N*; Table 5), indicating that blockade of EAAT2 but not EAAT5 makes RBs more depolarized in response to light stimulation than the control condition; this is in contrast to the traditional view which attributes the effect of TBOA on RB membrane potentials to blocking EAAT5-mediated Cl conductance (Veruki et al., 2006).

### EAAT1/GLAST in Müller cells does not influence neurotransmission at RB→AII synapses

**Figure 6.**
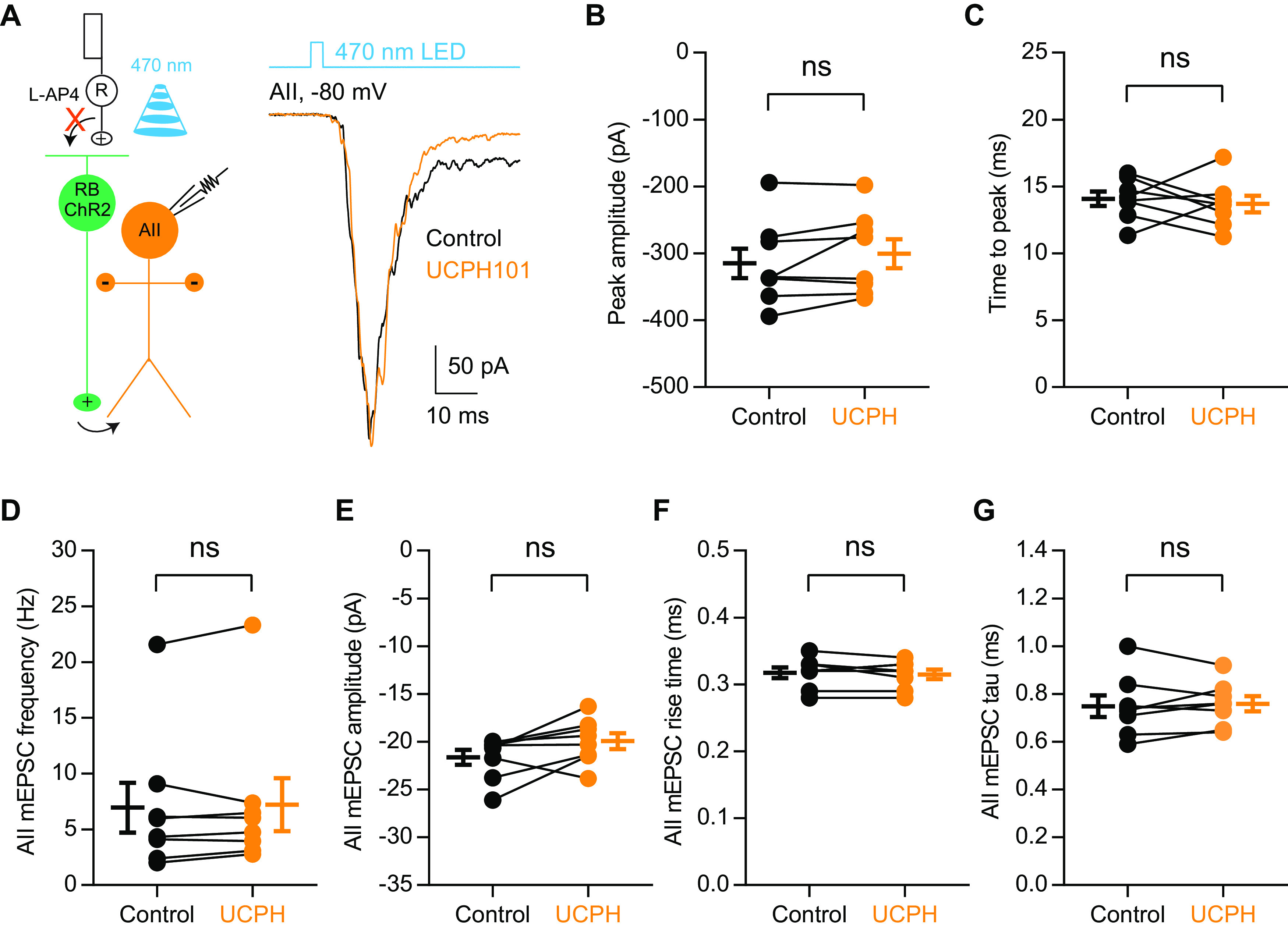
EAAT1 in Müller cells does not influence neurotransmission at RB→AII synapses. ***A***, The EPSCs recorded in AII amacrine cells, which were evoked by activating ChR2+ RBs with 470-nm LED light stimulation, were not affected by 50 μm UCPH101, a selective blocker of EAAT1 expressed exclusively in Müller cells. V_hold_ = −80 mV. R, rod; RB, rod bipolar cell; ChR2, channelrhodopsin-2. ***B***, ***C***, UCPH101 did not change the peak amplitude or time to peak of ChR2-evoked EPSCs (*n* = 8). ***D–G***, UCPH101 did not influence the frequency, amplitude, rise time, or tau of mEPSCs recorded in AIIs (*n* = 8). mEPSCs, miniature EPSCs. The data were represented as mean ± SEM. Wilcoxon signed-rank test or Student’s *t* test was used where appropriate. ns, not significantly different. See also Table 6.

EAAT1 (also called GLAST), the glutamate transporter exclusively expressed in Müller glial cells, is thought to be responsible for the major retinal glutamate uptake (Derouiche and Rauen, 1995; Rauen et al., 1996; Harada et al., 1998; Pow et al., 2000; Fyk-Kolodziej et al., 2004). Genetic deletion, antisense knock-down or pharmacological blockade of GLAST in mouse or rat retinas severely compromises the b-wave amplitudes of dark-adapted electroretinograms (ERGs), suggesting that GLAST in Müller cells might be involved in regulating the activities of RBs (Harada et al., 1998; Barnett and Pow, 2000; Tse et al., 2014). To determine whether GLAST may be involved in regulating neurotransmission at RB→AII synapses, we recorded ChR2-evoked EPSCs in AIIs and tested the effect of UCPH101, a selective GLAST blocker. Application of 50 μm UCPH101, however, did not have any significant effect on the peak amplitude or time to peak of EPSCs (Fig. 6*A–C*; Table 6).

**Table 6:** The effects of UCPH101 on AII responses

	Data	Data structure	Type of test	Power	Mean ± SEM	Number of cells
	Effect of UCPH on AII EPSC amplitude					
	Control	Normal distribution			−315.00 ± 22.05	8
	50 μm UCPH	Normal distribution			−300.60 ± 21.52	8
a	Control vs UCPH		Paired Student’s *t* test	*p* = 0.1588		8
	Effect of UCPH on AII EPSC time to peak (unit: ms)					
	Control	Normal distribution			14.08 ± 0.53	8
	50 μm UCPH	Normal distribution			13.69 ± 0.63	8
b	Control vs UCPH		Paired Student’s *t* test	*p* = 0.6306		8
	Effect of UCPH on AII mEPSC frequency (unit: Hz)					
	Control	Normal distribution			6.95 ± 2.24	8
	50 μm UCPH	Normal distribution			7.22 ± 2.37	8
c	Control vs UCPH		Paired Student’s *t* test	*p* = 0.4685		8
	DHK effect on AII mEPSC amplitude (unit: pA)					
	Control	Normal distribution			−21.62 ± 0.78	8
	50 μm UCPH	Normal distribution			−19.94 ± 0.82	8
d	Control vs UCPH		Paired Student’s *t* test	*p* = 0.0707		8
	Effect of UCPH on AII mEPSC rise time (unit: ms)					
	Control	Non-normal distribution			0.32 ± 0.01	8
	50 μm UCPH	Normal distribution			0.32 ± 0.01	8
e	Control vs UCPH		Wilcoxon signed-rank test	*p* = 0.8125		8
	Effect of UCPH on AII mEPSC tau (unit: ms)					
	Control	Normal distribution			0.75 ± 0.04	8
	50 μm UCPH	Normal distribution			0.76 ± 0.03	8
f	Control vs UCPH		Paired Student’s *t* test	*p* = 0.6382		8

UCPH101, a selective EAAT1/GLAST blocker; AII, AII amacrine cell.

In addition, UCPH101 did not affect the frequency, amplitude, rise time, or tau of mEPSCs recorded in AIIs (Fig. 6*D–G*; Table 6), indicating that blockade of GLAST in Müller cells does not influence spontaneous release from RB terminals.

These results therefore demonstrated that glutamate uptake by Müller cells does not influence synaptic transmission from RB terminals.

### EAAT5 plays a predominant role in regulating neurotransmission at RB→AII synapses

**Figure 7.**
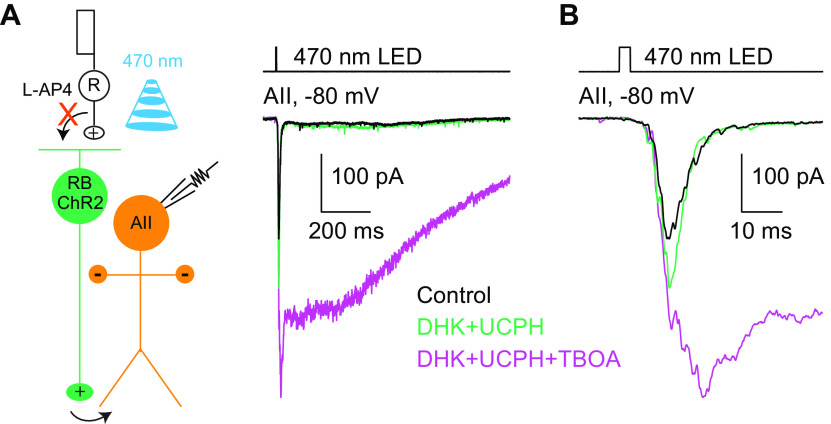
EAAT5 plays a predominant role in regulating neurotransmission at RB→AII synapses. ***A***, The EPSCs recorded in AII amacrine cells, which were evoked by 470-nm LED light stimulation of ChR2-expressing RBs, were increased slightly by co-application of DHK (200 μm) and UCPH101 (50 μm; *n* = 8), selective blockers of EAAT2 and EAAT1, respectively, and then enhanced more strongly by application of TBOA (50 μm; *n* = 3), a nonselective blocker of all EAATs. V_hold_ = −80 mV. R, rod; RB, rod bipolar cell; ChR2, channelrhodopsin-2. ***B***, Magnification of the traces shown in ***A***.

The considerably stronger effect of TBOA than DHK and UCPH101 on ChR2-evoked EPSCs in AIIs led us to the hypothesis that EAAT5 played a predominant role in regulating synaptic transmission at RB→AII synapses. To test this hypothesis, we examined the effect of TBOA on ChR2-evoked EPSCs under the experimental condition in which EAAT2 and GLAST were blocked by DHK and UCPH101. In general, co-application of 200 μm DHK and 50 μm UCPH101 (*n* = 8) generated similar effect to that of DHK applied alone on AII EPSCs (*n* = 7); and in the presence of DHK and UCPH101, application of TBOA enhanced both the transient and sustained components of EPSCs significantly (*n* = 3; Fig. 7).

Taken together, these results proved our hypothesis that EAAT5 plays a predominant role in regulating synaptic transmission at RB→AII synapses.

### Blockade of presynaptic EAAT5 but not EAAT2 reduces temporal resolution at RB→AII synapses

A recent study with EAAT5 knock-out mice has revealed an important role of EAAT5 for improving temporal resolution in the mouse retina, especially under mesopic light conditions (Gehlen et al., 2021). It is likely that genetic ablation of EAAT5 primarily affects signal transmission in the rod bipolar pathway (Gehlen et al., 2021), which functions under both scotopic and mesopic conditions (Ke et al., 2014). Because rods and RBs, both of which are essential neurons in the rod bipolar pathway, express EAAT5, the exact sites that may influence temporal resolution, however, remain to be determined.

**Figure 8.**
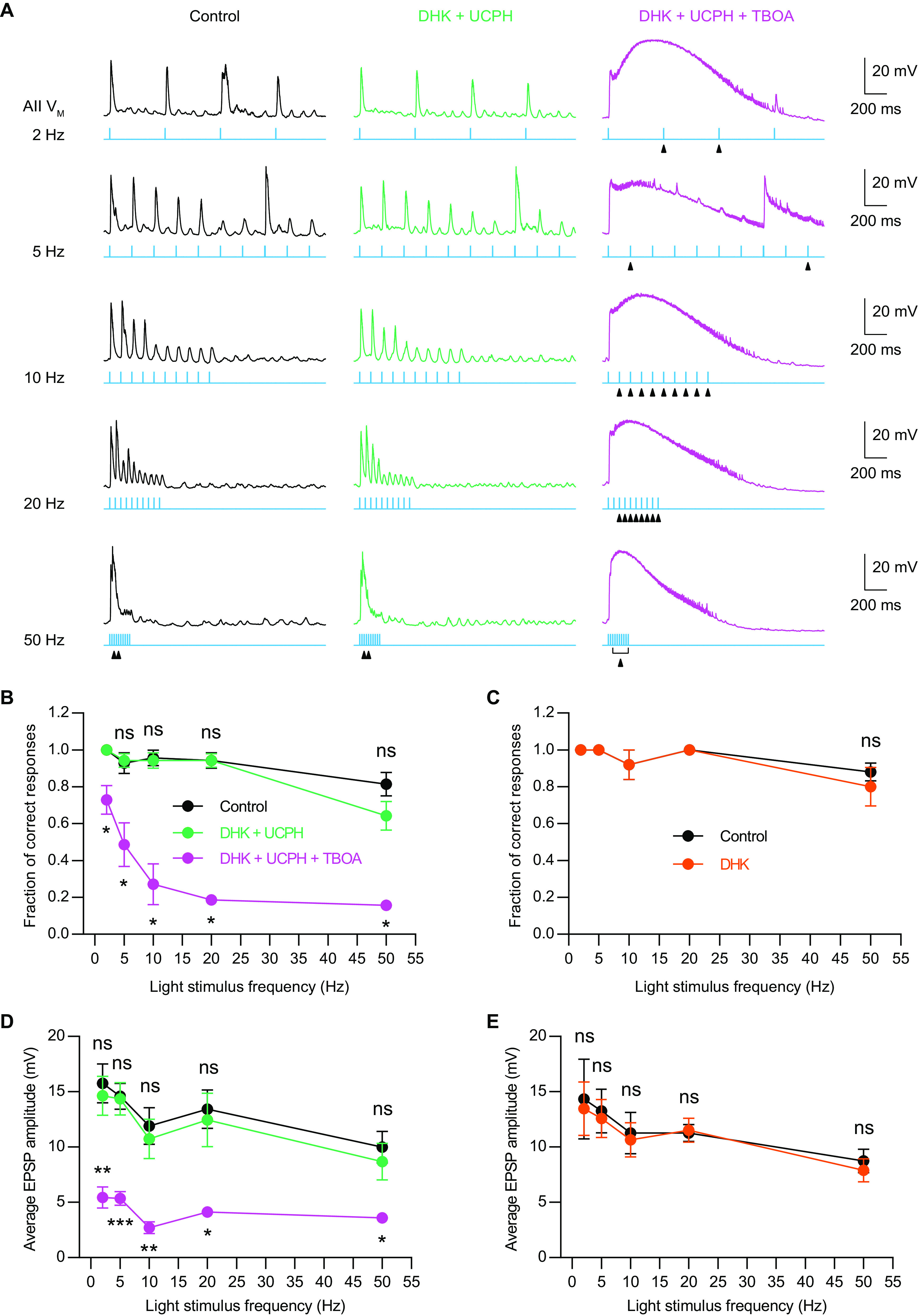
Blockade of presynaptic EAAT5 but not EAAT2 reduces temporal resolution at RB→AII synapses. ***A***, Representative traces showing the EPSPs recorded in AII amacrine cells, which were evoked by activating ChR2-expressing RBs with 470-nm LED light stimulation. The frequencies of light stimulation were in the range of 2–50 Hz. Under the control condition, the membrane potentials of AIIs could follow the 10 consecutive flashes very well even at stimulus frequency as high as 25 Hz (left panel). Co-application of 50 μm UCPH101, a selective EAAT1 blocker, and 200 μm DHK, a selective EAAT2 blocker, did not significantly influence AIIs’ responses to flashes (middle panel). But in the presence of 50 μm TBOA, a nonselective blocker of EAATs, AIIs failed to response to some individual flashes (marked by triangles; right panel), especially at stimulus frequencies higher than 10 Hz. ***B***, Summary data showing the fractions of correct responses for AIIs under three different experimental conditions. The fraction of correct responses was plotted as a function of light stimulus frequency. Application of 50 μm TBOA significantly reduced the fraction of correct responses at various stimulus frequencies (*n* = 7). ***C***, Summary data showing the fractions of correct responses for AIIs under control and DHK conditions. Application of 200 μm DHK did not significantly change the fraction of correct responses (*n* = 5). ***D***, Summary data showing the average amplitudes of AII EPSPs under three different experimental conditions. The average EPSP amplitude was plotted as a function of light stimulus frequency. Application of 50 μm TBOA significantly reduced the average EPSP amplitude at various stimulus frequencies (*n* = 7). ***E***, Summary data showing the average amplitudes of AII EPSPs under control and DHK conditions. Application of 200 μm DHK did not significantly change the average EPSP amplitude (*n* = 5). The data were represented as mean ± SEM. Wilcoxon signed-rank test or paired *t* test was used for comparison. *p < 0.05, **p < 0.01, ***p < 0.001; ns, not significantly different. See also Tables 7 and 8.

We recorded EPSPs from AIIs, which were evoked by activating ChR2-expressing RBs with a stimulus train of 10 consecutive flashes at various frequencies, and tested the effects of EAAT-related drugs. The frequencies of light stimulation were in the range of 2–50 Hz. Under the control condition, EPSPs recorded in AIIs could follow the individual flashes very well even at the stimulus frequency as high as 25 Hz; at the stimulus frequency of 50 Hz, there was relatively high possibility that AIIs only responded to some, but not all, flashes in a stimulus train (five of seven cells; Fig. 8*A*,*B*; Table 7). Blockade of EAAT1 and EAAT2 by co-application of 50 μm UCPH101 and 200 μm DHK did not make significant differences (Fig. 8*A*,*B*; Table 7). Under the experimental condition with subsequent application of 50 μm TBOA to block EAAT5 in RBs, however, AIIs failed to response to some individual flashes, and at the stimulus frequencies of 10 Hz and higher, AIIs were able to response to the first one or two flashes only (Fig. 8*A*,*B*; Table 7). In contrast, blockade of EAAT2 by application of 200 μm DHK alone did not influence AIIs’ responses to flashes at various frequencies (Fig. 8*C*; Table 7).

**Table 7:** The effects of EAAT-related drugs on the fraction of correct responses in AIIs at various light stimulus frequencies

	Data	Data structure	Type of test	Power	Mean ± SEM	Number of cells
	Effects of DHK, UCPH101 and TBOA on the fraction of correct responses in AIIs at various stimulus frequencies					
	2-Hz stimulus frequency					
	Control	Non-normal distribution			1.00 ± 0.00	7
	200 μm DHK + 50 μm UCPH101	Non-normal distribution			1.00 ± 0.00	7
	200 μm DHK + 50 μm UCPH101 + 50 μm TBOA	Normal distribution			0.73 ± 0.08	7
a	Control vs DHK + UCPH		Wilcoxon signed-rank test	N/A		7
b	DHK + UCPH vs DHK + UCPH + TBOA		Wilcoxon signed-rank test	*p* = 0.0313		7
	5-Hz stimulus frequency					
	Control	Non-normal distribution			0.93 ± 0.06	7
	200 μm DHK + 50 μm UCPH101	Non-normal distribution			0.94 ± 0.04	7
	200 μm DHK + 50 μm UCPH101 + 50 μm TBOA	Normal distribution			0.49 ± 0.12	7
c	Control vs DHK + UCPH		Wilcoxon signed-rank test	*p* > 0.9999		7
d	DHK + UCPH vs DHK + UCPH + TBOA		Wilcoxon signed-rank test	*p* = 0.0313		7
	10-Hz stimulus frequency					
	Control	Non-normal distribution			0.96 ± 0.04	7
	200 μm DHK + 50 μm UCPH101	Non-normal distribution			0.94 ± 0.04	7
	200 μm DHK + 50 μm UCPH101 + 50 μm TBOA	Non-normal distribution			0.27 ± 0.11	7
e	Control vs DHK + UCPH		Wilcoxon signed-rank test	*p* > 0.9999		7
f	DHK + UCPH vs DHK + UCPH + TBOA		Wilcoxon signed-rank test	*p* = 0.0156		7
	20-Hz stimulus frequency					
	Control	Non-normal distribution			0.94 ± 0.04	7
	200 μm DHK + 50 μm UCPH101	Non-normal distribution			0.94 ± 0.04	7
	200 μm DHK + 50 μm UCPH101 + 50 μm TBOA	Normal distribution			0.19 ± 0.03	7
g	Control vs DHK + UCPH		Wilcoxon signed-rank test	*p* > 0.9999		7
h	DHK + UCPH vs DHK + UCPH + TBOA		Wilcoxon signed-rank test	*p* = 0.0156		7
	50-Hz stimulus frequency					
	Control	Normal distribution			0.81 ± 0.06	7
	200 μm DHK + 50 μm UCPH101	Normal distribution			0.64 ± 0.08	7
	200 μm DHK + 50 μm UCPH101 + 50 μm TBOA	Non-normal distribution			0.16 ± 0.03	7
i	Control vs DHK + UCPH		Paired *t* test	*p* = 0.0533		7
j	DHK + UCPH vs DHK + UCPH + TBOA		Wilcoxon signed-rank test	*p* = 0.0156		7
	
	Effects of DHK alone on the fraction of correct responses in AIIs at various stimulus frequencies					
	2-Hz stimulus frequency					
	Control	Non-normal distribution			1.00 ± 0.00	5
	200 μm DHK	Non-normal distribution			1.00 ± 0.00	5
k	Control vs DHK		Wilcoxon signed-rank test	N/A		5
	5-Hz stimulus frequency					
	Control	Non-normal distribution			1.00 ± 0.00	5
	200 μm DHK	Non-normal distribution			1.00 ± 0.00	5
l	Control vs DHK		Wilcoxon signed-rank test	N/A		5
	10-Hz stimulus frequency					
	Control	Non-normal distribution			0.92 ± 0.08	5
	200 μm DHK	Non-normal distribution			0.92 ± 0.08	5
m	Control vs DHK		Wilcoxon signed-rank test	N/A		5
	20-Hz stimulus frequency					
	Control	Non-normal distribution			1.00 ± 0.00	5
	200 μm DHK	Non-normal distribution			1.00 ± 0.00	5
n	Control vs DHK		Wilcoxon signed-rank test	N/A		5
	50-Hz stimulus frequency					
	Control	Non-normal distribution			0.88 ± 0.05	5
	200 μm DHK	Normal distribution			0.80 ± 0.10	5
o	Control vs DHK		Wilcoxon signed-rank test	*p* = 0.5000		5

TBOA, a non-selective EAAT blocker; UCPH101, a selective EAAT1/GLAST blocker; DHK, a selective EAAT2 blocker; AII, AII amacrine cell; N/A, not applicable.

**Table 8:** The effects of EAAT-related drugs on the average amplitude of AII EPSPs at various light stimulus frequencies

	Data	Data structure	Type of test	Power	Mean ± SEM	Number of cells
	Effects of DHK, UCPH101 and TBOA on the average amplitude of AII EPSPs at various stimulus frequencies					
	2-Hz stimulus frequency					
	Control	Normal distribution			15.75 ± 1.77	7
	200 μm DHK + 50 μm UCPH101	Normal distribution			14.62 ± 1.77	7
	200 μm DHK + 50 μm UCPH101 + 50 μm TBOA	Normal distribution			5.44 ± 0.97	7
a	Control vs DHK + UCPH		Paired *t* test	*p* = 0.1704		7
b	DHK + UCPH vs DHK + UCPH + TBOA		Paired *t* test	*p* = 0.0013		7
	5-Hz stimulus frequency					
	Control	Normal distribution			14.58 ± 1.16	7
	200 μm DHK + 50 μm UCPH101	Normal distribution			14.36 ± 1.48	7
	200 μm DHK + 50 μm UCPH101 + 50 μm TBOA	Normal distribution			5.35 ± 0.62	7
c	Control vs DHK + UCPH		Paired *t* test	*p* = 0.7810		7
d	DHK + UCPH vs DHK + UCPH + TBOA		Paired *t* test	*p* = 0.0007		7
	10-Hz stimulus frequency					
	Control	Normal distribution			11.90 ± 1.66	7
	200 μm DHK + 50 μm UCPH101	Normal distribution			10.74 ± 1.79	7
	200 μm DHK + 50 μm UCPH101 + 50 μm TBOA	Normal distribution			2.70 ± 0.52	7
e	Control vs DHK + UCPH		Paired *t* test	*p* = 0.0746		7
f	DHK + UCPH vs DHK + UCPH + TBOA		Paired *t* test	*p* = 0.0054		7
	20-Hz stimulus frequency					
	Control	Normal distribution			13.41 ± 1.74	7
	200 μm DHK + 50 μm UCPH101	Normal distribution			12.45 ± 2.42	7
	200 μm DHK + 50 μm UCPH101 + 50 μm TBOA	Normal distribution			4.12 ± 0.29	7
g	Control vs DHK + UCPH		Paired *t* test	*p* = 0.4964		7
h	DHK + UCPH vs DHK + UCPH + TBOA		Paired *t* test	*p* = 0.0116		7
	50-Hz stimulus frequency					
	Control	Normal distribution			9.99 ± 1.42	7
	200 μm DHK + 50 μm UCPH101	Normal distribution			8.67 ± 1.65	7
	200 μm DHK + 50 μm UCPH101 + 50 μm TBOA	Non-normal distribution			3.58 ± 0.24	7
i	Control vs DHK + UCPH		Paired *t* test	*p* = 0.0763		7
j	DHK + UCPH vs DHK + UCPH + TBOA		Wilcoxon signed-rank test	*p* = 0.0156		7
	
	Effects of DHK alone on the average amplitude of AII EPSPs at various stimulus frequencies					
	2-Hz stimulus frequency					
	Control	Normal distribution			14.33 ± 3.61	5
	200 μm DHK	Normal distribution			13.46 ± 2.41	5
k	Control vs DHK		Paired *t* test	*p* = 0.6330		5
	5-Hz stimulus frequency					
	Control	Normal distribution			13.25 ± 1.97	5
	200 μm DHK	Normal distribution			12.57 ± 1.71	5
l	Control vs DHK		Paired *t* test	*p* = 0.5547		5
	10-Hz stimulus frequency					
	Control	Normal distribution			11.26 ± 1.87	5
	200 μm DHK	Normal distribution			10.65 ± 1.55	5
m	Control vs DHK		Paired *t* test	*p* = 0.4382		5
	20-Hz stimulus frequency					
	Control	Normal distribution			11.26 ± 0.76	5
	200 μm DHK	Normal distribution			11.51 ± 1.08	5
n	Control vs DHK		Paired *t* test	*p* = 0.8054		5
	50-Hz stimulus frequency					
	Control	Normal distribution			8.74 ± 1.07	5
	200 μm DHK	Normal distribution			7.88 ± 1.05	5
o	Control vs DHK		Paired *t* test	*p* = 0.0780		5

TBOA, a non-selective EAAT blocker; UCPH101, a selective EAAT1/GLAST blocker; DHK, a selective EAAT2 blocker; AII, AII amacrine cell.

The AII responses to individual flashes were also quantified by measuring the trough to peak EPSP amplitudes. Because the EPSP amplitudes varied greatly (Fig. 8*A*), we used the average amplitude of AII EPSPs in response to a stimulus train of 10 flashes for comparisons between different experimental conditions. Under the control condition, the average EPSP amplitude seemed to decrease gradually as the light stimulus frequency increased, albeit with a sag at the stimulus frequency of 10 Hz (Fig. 8*D*,*E*). Application of DHK alone or co-application of UCPH101 and DHK did not change the average EPSP amplitude significantly, but subsequent application of TBOA strongly reduced it (Fig. 8*D*,*E*; Table 8).

In conclusion, these results demonstrated that EAAT5 but not EAAT2 in RBs plays an important role in improving temporal resolution at RB→AII synapses.

## Discussion

We combined molecular and functional analyses to determine the expression profiles, subcellular localization and functional roles of EAATs in mouse RBs. We found that EAAT2, together with the recently reported EAAT5 (Gehlen et al., 2021), are co-expressed in RB axon terminals near the release sites, thus revealing a common strategy (i.e., co-expression of EAAT2 and EAAT5) for the first (rod→RB synapse) and second (RB→AII synapse) visual synapses that function under dim light conditions. Additionally, we demonstrated that EAAT2 and EAAT5 shape synaptic transmission from RB terminals in significantly different ways, likely via distinct mechanisms. Although expression of EAAT2 has been extensively studied in BCs of various species since 1990s, the present work, to the best of our knowledge, is the first report about the function of EAAT2 in the inner retina. Finally, we also found that EAAT5 but not EAAT2 plays a predominant role in regulating temporal resolution at RB→AII synapses.

### The function of EAAT2 in the retina

It has been well established that EAAT2 primarily serves as an astroglial glutamate transporter in the CNS (Tzingounis and Wadiche, 2007; Danbolt et al., 2016; Rodríguez-Campuzano and Ortega, 2021). In the mammalian retina, however, EAAT2 is mainly expressed in the neurons such as photoreceptors and BCs. Although the expression patterns of EAAT2, including its splice variants GLT-1α, GLT-1B, and GLT-1v, have been studied extensively in the retinas of various species (Rauen and Kanner, 1994; Rauen et al., 1996, 2004; Eliasof et al., 1998b; Harada et al., 1998; Haverkamp and Wässle, 2000; Vandenbranden et al., 2000; Reye et al., 2002a, b; Fyk-Kolodziej et al., 2004; Niklaus et al., 2017), our current knowledge about the function of EAAT2 in retinal neurons is still limited. In lower vertebrates such as salamander, goldfish and zebrafish, EAAT2 in cone photoreceptors can modify the postsynaptic responses in horizontal cells or BCs (Gaal et al., 1998; Vandenbranden et al., 1996; Niklaus et al., 2017), whereas in mouse, EAAT2 does not influence neurotransmission at rod→RB synapse (Hasegawa et al., 2006). Also, genetic ablation or pharmacological blockade of EAAT2 in the mouse retina does not have any significant effect on the a-wave and b-wave amplitudes of dark-adapted ERGs, which reflect the activities of rod photoreceptors and RBs, respectively (Harada et al., 1998; Tse et al., 2014). However, previous studies have shown the presence of EAAT2 in rod and cone photoreceptors of mouse, rat and human by immunohistochemistry (Rauen and Kanner, 1994; Haverkamp and Wässle, 2000; Rauen et al., 2004), and in this study our scRNA-seq analysis with mouse rod and cone transcriptomes detected EAAT2 mRNAs at reasonable levels (Fig. 1*B*). The functions of EAAT2 in mammalian rod and cone photoreceptors therefore remain to be further explored.

EAAT2 is found to be expressed in certain types of BCs in almost all the species studied. To date, however, no published studies have reported the function of EAAT2 in BCs. In the present work, we found that in the mouse retina, EAAT2 was expressed in all BC types while coexisting with EAAT5 in RBs and some cone BCs (Figs. 1, 2). Functionally, we showed that blockade of EAAT2 in RB axon terminals enhanced and accelerated the postsynaptic responses in AIIs, likely by increasing the light-evoked voltage changes in RBs (Figs. 3, 4). Thus, in this study, we presented the first evidence that EAAT2 functions at BC ribbon synapses. Also, based on the distinct subcellular localizations of EAAT2 in different BC types (Fig. 3*A*; e.g., expression at the somata vs expression in the axon terminals), we could imagine that EAAT2 may play different roles in various BCs.

### The function of EAAT5 in the retina

EAAT5 is originally known as a retina-specific glutamate transporter (Arriza et al., 1997), but recent studies also have reported that it is widely distributed in non-neuronal tissues (Lee et al., 2013). In the mouse and rat retinas, EAAT5 is mainly expressed in rod and cone photoreceptors as well as in BCs, including RBs (Pow and Barnett, 2000; Wersinger et al., 2006).

To date, functional studies about EAAT5 in the retina have been focused on the rod bipolar pathway which consists of both rod and RB, probably because of its strong immunolabeling at photoreceptor and RB axon terminals. At mouse rod→RB synapses, EAAT5 plays a predominant role in regulating synaptic transmission: blockade of EAAT5 enhances and prolongs the postsynaptic responses of RBs (Hasegawa et al., 2006). Model simulations suggest that EAAT5 proteins densely packed near the release sites in rod terminals are essential for rapid removal of glutamate within the synaptic cleft (Hasegawa et al., 2006). At mouse and rat RB terminals, however, EAAT5 is generally thought to function as a large-conductance, glutamate-activated chloride channel; once activated by glutamate released from RBs, EAAT5 may contribute feedback inhibition to RB terminals and thus control the RB output (Arriza et al., 1997; Veruki et al., 2006; Wersinger et al., 2006; Gameiro et al., 2011; Schneider et al., 2014; Fahlke et al., 2016; Bligard et al., 2020; Lukasiewcz et al., 2021; Kovermann et al., 2022). Paired recordings of synaptically connected mouse RBs and AIIs have shown that pharmacological blockade of EAATs by TBOA influences the postsynaptic responses in AIIs, presumably by inhibiting the EAAT5-mediated Cl conductance and enhancing the membrane depolarizations in RBs (Veruki et al., 2006).

It is noteworthy, however, that previous observations of EAAT5 function in RBs rely mainly on the assumption that EAAT5 was the only subtype of EAATs expressed in the axon terminals of RBs. It is, then, quite striking that in our present work we found that EAAT2 also was expressed in RB axon terminals near release sites (Figs. 1-3). Therefore, those data achieved by previous studies for “EAAT5” function in RB terminals have to be interpreted very carefully.

We also found that the light-evoked depolarizations of RBs enhanced by DHK and TBOA were not significantly different (Figs. 4, 5), indicating that blockade of Cl conductance mediated by EAAT2 but not EAAT5 enhances the voltage changes in mouse RBs. In contrast, the effect of TBOA on the voltage changes in rat RBs is supposed to be mediated by blockade of EAAT5 (Veruki et al., 2006), mainly because of the observation of EAAT5 expression in the axon terminals of RBs by immunohistochemistry (Pow and Barnett, 2000). Because EAAT5 is generally thought to have a higher Cl conductance than EAAT2 (Schneider et al., 2014; Fahlke et al., 2016; Lukasiewcz et al., 2021; Kovermann et al., 2022), it was quite surprising that blockade of both EAAT2 and EAAT5 by TBOA did not generate a larger change in RB membrane potentials than blockade of EAAT2 alone by DHK (Fig. 5*N*). Nevertheless, it is worth noting that TBOA induced a large AHP following the fast depolarization of each RB (Fig. 5*L*). Thus, there exists the possibility that the putative effect of blocking EAAT5-mediated Cl conductance by TBOA on RB voltage change is partially counteracted by the AHP. The origin of the AHP, however, is still unknown. In the presence of TBOA, excess glutamate might activate metabotropic glutamate receptors in RBs, which would in turn enhance the activities of G protein-activated K^+^ (GIRK) channels (Saugstad et al., 1996; Sharon et al., 1997; Knoflach and Kemp, 1998) or small conductance Ca^2+^-activated K^+^ (SK) channels (Sourdet et al., 2003; García-Negredo et al., 2014), and thereby induce the AHPs. The effect of TBOA on membrane potentials of RBs apparently could not account for its large, prolonged effect on AII responses (Fig. 5*A*,*L*), suggesting that blockade of EAAT5 regulates neurotransmission at RB→AII synapses presumably by blocking its ability of glutamate transport and/or glutamate buffering (Tzingounis and Wadiche, 2007), but not by blocking EAAT5-mediated Cl conductance.

Recently, a new EAAT5 knock-out mouse model has been generated and used to study the functional role(s) of EAAT5, and consequently, EAAT5 is found to be important for improving temporal resolution in the retina, probably by affecting the rod bipolar pathway in the mesopic range (Gehlen et al., 2021). In the present study, we demonstrated that pharmacological blockade of EAAT5 but not EAAT2 influenced temporal resolution at mouse RB→AII synapses (Fig. 8). However, since the rod bipolar pathway operates under both scotopic and mesopic illuminations (Ke et al., 2014), we cannot differentiate between the two light levels under our experimental conditions. Given the similar effects of TBOA (both enhance and prolong the postsynaptic responses) on neurotransmission at rod→RB synapses (Hasegawa et al., 2006) and at RB→AII synapses (Fig. 5), we could imagine that pharmacological blockade of EAAT5 also would influence temporal resolution at rod→RB synapses.

### Co-expression of EAAT2 and EAAT5 in retinal neurons

Accumulating evidence from immunohistochemical studies has shown that EAAT2 and EAAT5 are very likely to coexist in rod and cone photoreceptors. Whether EAAT2 and EAAT5 may be co-expressed in any certain type of BCs, however, is unknown. In the present work, we found that EAAT2 and EAAT5 coexisted in RBs as well as in types 3B, 4, 5B, and 5C BCs (Fig. 1). Co-expression of EAAT2 and EAAT5, therefore, seems to be a common strategy used by both first-order and second-order retinal neurons. It will, then, be interesting to explore in future studies why some BC types express both subtypes of EAATs while others express EAAT2 only.

In the present work, we also found that EAAT2 and EAAT5 differentially shape signal transmission at RB→AII synapses, presumably via distinct mechanisms (Figs. 4, 5, 8). Generally, blocking EAAT5 likely has a stronger effect on retinal signal transmission, this may explain the observations from previous studies showing that genetic ablation or pharmacological blockade of EAAT2 does not significantly influence the a-wave or b-wave amplitudes of dark-adapted ERGs (Harada et al., 1998; Tse et al., 2014).

### The function of EAAT1/GLAST in retinal Müller cells

GLAST, which is exclusively expressed in Müller cells, is thought to be the major glutamate sink in the retina, this traditional notion is largely drawn from the observations in autoradiographic uptake studies in which exogenously-applied radiolabeled glutamate is absorbed mainly by Müller cells (Derouiche and Rauen, 1995; Rauen et al., 1996, 1998; Pow et al., 2000). In addition, genetic ablation, antisense knock-down or pharmacological blockade (i.e., by UCPH101) of GLAST in rodents severely reduces the b-wave amplitudes of dark-adapted ERGs, suggesting that GLAST in Müller cells might be involved in regulating the activities of RBs (Harada et al., 1998; Barnett and Pow, 2000; Tse et al., 2014).

However, there are also some electrophysiological studies arguing that glutamate uptake by Müller cells is not a fast and efficient process for regulating synaptic transmission (Gaal et al., 1998; Hasegawa et al., 2006; Rowan et al., 2010). The electrically evoked RB responses in GLAST knock-out mice were not different from those in wild-type mice, indicating that GLAST in Müller cells may not play a predominant role at rod→RB synapses (Hasegawa et al., 2006). Consistent with these results, in the present study, we provided direct evidence that blockade of GLAST in Müller cells does not influence neurotransmission at RB→AII synapses (Fig. 6). Therefore, it would seem likely that GLAST in Müller cells plays a predominant role in removing glutamate diffused out of the synaptic cleft to prevent excitotoxicity, but a less important role in regulating synaptic transmission directly.

## Synthesis

Reviewing Editor: Katalin Toth, University of Ottawa Decisions are customarily a result of the Reviewing Editor and the peer reviewers coming together and discussing their recommendations until a consensus is reached. When revisions are invited, a fact-based synthesis statement explaining their decision and outlining what is needed to prepare a revision will be listed below. The following reviewer(s) agreed to reveal their identity: NONE.

The present study provides novel data on the role of EAAT2 and EAAT5 in synaptic transmission in rod bipolar cells. The study is generally well planned and executed. However, both reviewers raised several issues that would require the authors’ attention. These are largely focused on proper controls and statistical approaches. Addressing the issues detailed in the reviewers’ comments will undoubtedly increase the quality of this interesting study.

Rev. #1.

In this manuscript, the authors report that the glutamate transporters EAAT2 and EAAT5 differentially modulate synaptic transmission from rod bipolar cell terminals. Using a combination of single-cell RNA sequencing and immunohistochemistry, the authors first showed localized expression of EAAT2 and EAAT5 in the presynaptic terminals of rod bipolar cells (RB). Notably, while EAAT2 was largely expressed across groups of cells, EAAT5 co-expression was rare and a feature of RB. Second, the authors used electrophysiology and optogenetic to dissect the function of EAAT2 and EAAT5 in the rod bipolar cells terminals. They uncovered that EAAT2 and EAAT5 modulate synaptic transmission differentially at the RB to AII amacrine cell synapse. Whereas EAAT5 controlled the peak amplitude and the kinetics of postsynaptic excitatory potentials, EAAT2 had only a small effect on these parameters. Functionally, blockade of EAAT5 activity revealed that this transporter was important for proper signaling during repetitive activity. The results reported here are novel and the experiments appear to be well-executed. Below are comments and observations which dampened my enthusiasm for this paper.

1) The main conclusions of this paper rest on pharmacological dissection of EAAT’s activity. In Figure 7, the conclusions are based on the assumption that the effect of TBOA is purely on EAAT5, because EAAT1 and 2 are fully pre-blocked by UCPH101 and DHK compounds. A potential caveat is that EAAT1 and EAAT2 are not fully blocked at the concentrations used in the acute slices. In Figure 8, the authors compared the effects of TBOA and DHK. In particular, TBOA was used without pre-blocking EAAT1 and EEAT2. Therefore, the effect of TBOA in this case cannot be attributed to EAAT5 contrary to what the authors mention in the abstract.

2) The use of optogenetic techniques in the retina might be problematic due to the nature of the tissue, leading to non-specific stimulation. Did the authors ever perform control experiments in non-Ai32 animals (either in WT or Pcp2-GFP/YFP)?

3) The origin of miniature (mEPSCs) reported in Fig. 4H-K, Fig. 5H-K and Fig. 6D-G remains undetermined. While optogenetically-evoked EPSCs clearly originate from genetically-defined Pcp2 neurons, miniature EPSCs are thought to be Ca2+-independent events. The authors claim that mEPSCs recorded in the presence of drugs would have the same origin as optogenetically-evoked EPSCs. AII cells also receive inputs from OFF cone bipolar cells which could contribute to the mEPSCs recorded.

4) Statistical treatment of the data is not appropriate. The authors provide a clear summary of statistical test on the different datasets obtained, which should be commended. On the other hand, it is hard to understand why the data is sometimes normalized prior to performing statistical tests. This is generally inappropriate and can lead to misinterpretation of the data. I do not understand why the authors indicate that ‘For better comparison between different groups, data acquired from each cell were normalized to the value under control condition where appropriate’ in the Material and Methods section.

For example, Fig. 4D should be presented as in other panels in that figure, with the peak amplitude values before and after drug treatment. There is no comparison involved between different groups; in this example, the authors are testing for the effects of DHK or GT949 on peak amplitude values. Normalizing the data prior to statistical testing removes the intrinsic variance in the control data. This comment applies to all panels with statistical tests on normalized values.

5) Data presentation: for pairwise comparisons, data should be presented with lines linking individual neurons for the control and the drug treated condition.

6) In Figure 8, the authors analyze the ‘Fraction of correct responses’. What is defined as a correct response? The authors may want to consider using trough to peak measurements of EPSP amplitude as a more quantitative measure than correct responses. This would also alleviate the normalization issue and allow for statistical comparison of the data. In some instances, it is unclear what the data is normalized to. For example, why is the control 5 Hz 0.95 instead of 1 in Table 7? Data for DHK treatment also needs to be shown.

Rev. #2.

This study examines the function of the glutamate transporters EAAT2 and EAAT5 in the mouse retina. Novel aspects of the study include the analysis of co-expression of both glutamate transporters in the different bipolar cell types and immunohistochemical localization of EAAT2 in close proximity to the synaptic ribbon at rod bipolar cells.

The authors then perform a series of electrophysiological experiments recording from AII amacrine cells in retinal slices of mice that express ChR2 in rod bipolar cells comparing AII responses in the presence and absence of different EAAT blockers. Taken together, the study suggests that EAAT2 and EAAT5 located at rod bipolar synapses play different roles. The authors showed that blockade of EAAT2 enhanced and accelerated the postsynaptic responses in AIIs, while blocking of EAAT5 decreased temporal resolution.

I have a few suggestions to the authors to improve the manuscript. This includes providing additional information for the reader and additional discussion.

1. There are several EAAT2 splice variants known. The authors should discuss whether the antibody they used recognizes all of these variants or only a subset.

2. The authors stimulated ChR2 in rod bipolar cells and measured the response in postsynaptic AII amacrine cells, but of course light intensity for ChR2 activation cannot be related directly to the light regime under physiological conditions. This makes it difficult to compare the study to previous studies on EAAT5 function by Gehlen et al or Bligard et al. As the authors seem to have considerable experience with AII recordings, can they provide any clue whether their experimental conditions relate to the scotopic or (most likely) mesopic range? Gehlen et al showed that EAAT5 was more important in the upper mesopic range, probably because more glutamate is released with brighter stimuli. Bligard et al did not observe that. Did the authors test different light intensities for ChR2 stimulation that would help to clarify this issue?

3. The authors suggest that blockade of EAAT2 but not EAAT5 enhances the voltage changes in mouse RBs while blockade of EAAT5 regulates neurotransmission presumably by blocking its ability of glutamate transport and/or glutamate buffering but not by blocking EAAT5-mediated Cl conductance. As the authors state, these conclusions are in contrast to previous studies on the function of EAAT5 in the retina but also on the properties of EAATs in general. EAAT2 was shown to be a good transporter, but displayed weak chloride channel activity, while for EAAT5 it was the other way round. Can the authors discuss this issue further?

4. What is the origin of the AHP observed under TBOA block?

Minor points:

Line 335: please give here one or two citations that show that all transmission is blocked by that approach.

Line 387: why do the values rise? One might argue that blockade of a chloride channel increases the input resistance and thus accelerates changes in membrane voltage of the RB.

Line 478: “We found that EAAT2 and EAAT5 are co-expressed in RB axon terminals near the release sites ...”. This statement is not correct, as the authors only localized EAAT2, but not EAAT5. It would be better to say that from the results of Gehlen et al (EAAT5) and their own results (EAAT2), it can be concluded that both transporters are found in rod bipolar axon terminals close to the glutamate release sites. The authors state this in a similar way at line 327.

Line 524: “expression on the cell membrane vs. expression in the axon terminals ...”. Being a transporter, in both soma and axon terminal, EAAT2 should be in the cell membrane. I assume the authors want to distinguish the function at the soma from the function at the axon terminal. What could be the function of EAAT2 at the soma?

Line 923: how was the fraction of correct responses determined? Does that mean response to individual flashes or to the entire stimulus train? Sometimes the cells respond to only some, but not to all stimuli. Would that be considered an incorrect response? Please provide the rational in the method section.

In the figures, the label seems sometimes confusing. For example, in Fig 3 L, the control curve is black and the DHK curve is grey, yet in M DHK values are given as black dots. This may lead to confusion.
